# Fpk1/2 kinases regulate cellular sphingoid long-chain base abundance and alter cellular resistance to LCB elevation or depletion

**DOI:** 10.1002/mbo3.160

**Published:** 2014-02-10

**Authors:** Yukari Yamane-Sando, Etsuko Shimobayashi, Mitsugu Shimobayashi, Yasunori Kozutsumi, Shogo Oka, Hiromu Takematsu

**Affiliations:** 1 Laboratory of Membrane Biochemistry and Biophysics, Graduate School of Biostudies, Kyoto University46-29 Yoshida-shimoadachi, Sakyo, Kyoto, 606-8502, Japan; 2 Department of Biological Chemistry, Human Health Sciences, Graduate School of Medicine, Kyoto University53 Shogoin-Kawahara, Sakyo, Kyoto, 606-8507, Japan

**Keywords:** DIR screening, long-chain base, protein kinase, sphingolipids, yeast

## Abstract

Sphingolipids are a family of eukaryotic lipids biosynthesized from sphingoid long-chain bases (LCBs). Sphingolipids are an essential class of lipids, as their depletion results in cell death. However, acute LCB supplementation is also toxic; thus, proper cellular LCB levels should be maintained. To characterize the “sphingolipid-signaling intercross,” we performed a kinome screening assay in which budding yeast protein kinase-knockout strains were screened for resistance to ISP-1, a potent inhibitor of LCB biosynthesis. Here, one pair of such *DIR* (deletion-mediated ISP-1 resistance) genes, *FPK1* and *FPK2*, was further characterized. Cellular LCB levels increased in the *fpk1/2Δ* strain, which was hypersensitive to phytosphingosine (PHS), a major LCB species of yeast cells. Concomitantly, this strain acquired resistance to ISP-1. Fpk1 and Fpk2 were involved in two downstream events; that is, ISP-1 uptake due to aminophospholipid flippase and LCB degradation due to LCB4 expression. RSK3, which belongs to the p90-S6K subfamily, was identified as a functional counterpart of Fpk1/2 in mammalian cells as the *RSK3* gene functionally complemented the ISP-1-resistant phenotype of *fpk1/2Δ* cells.

## Introduction

Sphingolipids comprise a class of lipids that are biosynthesized from sphingoid long-chain bases (LCBs), such as phytosphingosine (PHS) (Fig. S1A; Dickson 1998). Given the importance of sphingolipids for membrane biology, the regulation of sphingolipid biosynthesis has attracted much attention in the field of cell biology (Liu et al. 2005), particularly in relation to cellular signaling under stress conditions, such as heat stress (Dickson et al. 1997; Jenkins et al. 1997; Cowart et al. 2003) in the yeast *Saccharomyces cerevisiae* (Buede et al. 1991; Nagiec et al. 1994). Sphingolipids may also act as signaling molecules in the control of the longevity of organisms (Huang et al. 2012). However, information regarding the signaling aspects of cellular sphingolipids remains largely fragmentary.

It has been suggested that LCBs, rather than complex sphingolipids, are required for survival of yeast cells (Schorling et al. 2001). However, it is not clear how a lack of LCBs leads to lethal consequences. Recent studies have indicated that endoplasmic reticulum stress could be induced in sphingolipid-depleted cells (Kajiwara et al. 2012). In heat-stressed cells, LCBs may function as lipid mediators to protect cells, controlling intracellular protein aggregation and translation (Meier et al. 2006; Cowart et al. 2010). Cross-talk between LCB biosynthetic components in cellular signaling has been investigated using mutant cells that lack the proper sphingolipid profiles (Zhao et al. 1994), as well as through the inhibition of cellular serine palmitoyltransferase using the natural fungal lipid ISP-1/myriocin (Kluepfel et al. 1972; Miyake et al. 1995).

Cellular maintenance of LCB levels is important because excess supplementation of PHS, a major LCB species in yeast, causes acute G_1_ arrest of the cell cycle and subsequent cell death (Dickson and Lester 2002). This cell cycle arrest could be related to LCB-mediated stress signaling upon exposure of cells to elevated temperatures (Dickson et al. 1997; Jenkins et al. 1997). Maintenance of proper LCB levels is regulated, at least in part, by cell signaling events. The serine/threonine protein kinase Ypk1/Sli2 (Sun et al. 2000) was shown to play a major role in sphingolipid homeostasis by phosphorylating Orm1/2 (Roelants et al. 2011). Moreover, Orm1/2 phosphorylation, acting in conjunction with the Pkh1/Ypk1 signaling cascade and Cdc55-containing PP2A protein phosphatase, may function in biological regulatory circuits; for example, in the heat stress response (Sun et al. 2012), during which LCB species such as PHS are transiently induced. In fact, a functional relationship between Ypk1 and LCBs was revealed in which YPK1 was found to be a multicopy repressor gene for ISP-1-mediated yeast lethality (Sun et al. 2000). However, relatively little is known regarding signaling events involving LCBs. Thus, further systematic analyses on cellular signaling events regarding cellular sphingolipids are required.

We probed the yeast “kinome” (Manning et al. 2002) with ISP-1 to systematically screen for yeast kinase genes that affect cell viability under conditions of sphingolipid biosynthesis inhibition; this kinome screening was designated as gene *d*eletion-mediated *I*SP-1 *r*esistance (*DIR*). We used this approach to increase our understanding of the interrelationships between protein kinases and sphingolipids, including regulation of sphingolipids by kinase(s) and vice versa. Using this method, we identified nine protein kinase genes, deletion of which resulted in resistance to ISP-1. The cellular LCB levels of each *DIR* mutant were assayed to categorize the mutants. One paralogous set of *DIR* kinases, *FPK1* and *FPK2*, was functionally analyzed in detail. Fpk1/2 protein kinases were originally reported as regulators of aminophospholipid flippase (Nakano et al. 2008), and subsequently proposed as substrates of Ypk1 (Roelants et al. 2010). We found that Fpk1/2 redundantly functioned to suppress basal LCB expression and control ISP-1 uptake. Thus, mutation of *FPK1/2* conferred concomitant resistance to ISP-1 and sensitization to PHS. Our data indicated that Fpk1/2 kinases play a role in maintaining appropriate cellular LCB levels. We also found that mammalian RSK3, which belongs to the p90-S6K kinase family, was a functional counterpart of Fpk1/2 based on the results of genetic complementation assays. These data are indicative of evolutionarily conserved signaling events, potentially involving cellular sphingolipids.

## Experimental Procedures

### Antibodies

The anti-Ypk1 antibody was reported previously (Tanoue et al. 2005). Antibodies against Fpk1 and Bap2 were raised by immunization of a rabbit six times at 1-week intervals with the recombinant N-terminal ∼100 amino acid residues conjugated to glutathione-*S*-transferase (GST) (MBL, Nagoya, Japan). Anti-Lcb4 rabbit antiserum was kindly provided by Dr. Akio Kihara (Hokkaido University). The anti-PGK1 antibody, HA, and GST were from Invitrogen (Life Technologies, Grand Island, NY), Covance (Princeton, NJ) and Upstate Biotechnology (Lake Placid, NY), respectively. The HRP-conjugated anti-mouse and anti-rabbit goat IgG were from Zymed (Life Technologies) and DAKO (Glostrup, Denmark), respectively.

### Yeast strains, culture conditions, and reagents

The yeast strains used in this study are listed in Table S3. Mutant strains and isogenic control strains were maintained on synthetic defined (SD) plates (Giaever et al. 2002). The conditions for cell culture were as reported previously (Kobayashi et al. 2005). Briefly, fresh colonies were inoculated into YPD medium and incubated overnight, unless otherwise stated. Overnight cultures of yeast cells were diluted to an optical density at 600 nm (OD_600_) of 0.2 in YPD medium and cultured. Cell growth was monitored by OD_600_ measurements. Cells in the logarithmic phase of growth were used. SR medium (6.7 g of yeast nitrogen base without amino acids [Difco, Detroit, MI] plus 20 g of raffinose per liter) was used for induction of the *GAL1* promoter. Duramycin (Sigma Chemical Co., St. Louis, MO) was dissolved in water. ISP-1/myriocin (Sigma) was dissolved in methanol, and PHS (Sigma) and stearylamine (Sigma) were dissolved in ethanol. Aureobasidin A and dihydrosphingosines were dissolved in ethanol and methanol, respectively. Cells were treated with PHS (20 *μ*M) and incubated for 15 or 30 min prior to harvesting. Treatment with ISP-1 (500–750 ng mL^−1^) was performed as described previously (Sun et al. 2000).

### Plasmid and yeast strain construction

Construction of the deletion strains was achieved through PCR-based homologous recombination, as described previously (Brachmann et al. 1998; Longtine et al. 1998). For strain *ypk1*^*S71A*^, a point mutation was introduced into *YPK1* in YEp351 by site-directed mutagenesis (Clontech Laboratories, Palo Alto, CA). The mutant *YPK1* fragment was then ligated into pFA6a-*HIS3MX6* to generate a “knock-in” plasmid. PCR-amplified *ypk1*^*S71A*^*-HISMX6* was transformed into BY4741 cells, and the transformants were selected for homologous recombination. To achieve gene overexpression, *FPK1* was cloned into YEp352. *FPK2* and *YPK1* were cloned into YEp351. *P*_*GAL1*_*-GST-YPK1* was kindly provided by Dr. Jason Ptacek (Yale University) (Zhu et al. 2000). For overexpression of GST-Fpk1/2 in yeast cells, a fragment that contained *GST-FPK1* and *GST-FPK2* was placed under the control of the *ADH1* promoter in YEp351 and YEp352, respectively, as described previously (Momoi et al. 2004). Human cDNA species for AGC kinases were purchased from the MGC Clone Collection (http://mgc.nci.nih.gov) and subcloned into the pDONR221 vector using the BP reaction. The YEp351 vector harboring the *ADH1* promoter and termination signal with destination cassette was used to target the LR reaction. The plasmid constructs and yeast strains used in the present study are listed in Tables S3 and S4 (Shimobayashi et al. 2010).

### DIR assay and other resistance assays

The expression of *d*eletion-mediated *I*SP-1 *r*esistance (*DIR*) genes were determined using one-by-one ISP-1 resistance spot assays with serial 1:5 dilutions of cells on YPD plates (Kobayashi et al. 2005). Briefly, each batch of commercially available kinase-knockout cells (Research Genetics, Huntsville, AL) (Table S1) was cultured in YPD medium overnight and diluted to an OD_600_ of 0.2 in prewarmed fresh medium for 4 h to obtain early log-phase cells. Cells were serially diluted and spotted onto YPD plates that contained ISP-1 (500–750 ng mL^−1^) or PHS (30 *μ*M) and incubated for 2–3 days. To avoid plate-by-plate variability, we included at least one set of wild-type (WT) control (BY4741) cells on each plate to evaluate resistance to ISP-1. We defined resistant (*DIR*) strains as those that grew more rapidly than the WT control when WT cells were strongly attenuated on ISP-1-containing plates. When SD plates were used elsewhere in this study, the cells were spotted onto agar medium that contained ISP-1 (500 ng mL^−1^) or PHS (20 *μ*M) and incubated for 3 days. To further evaluate ISP-1 resistance, OD_600_ values after 24-or 36-h liquid culture were also determined for strains identified as resistant by the plate-based assay.

### LCB measurement

Extraction and processing of LCBs from yeast cells for fluorescence high-performance liquid chromatography (HPLC) analysis using the AQC reagent (Waters, Milford, MS) were performed as described previously (Lester and Dickson 2001; Sun et al. 2012). Briefly, HPLC analysis was performed using a C18 column (4.6 × 250 mm, XDB-C18; Hewlett-Packard, Palo Alto, CA) on a Shimadzu LC10A series liquid chromatography system. Isocratic elution was carried out for 60 min at a flow rate of 1.0 mL min^−1^. Lipid-reacted AQC reagent was excited with 244-nm ultraviolet radiation, and the resultant emission signal at 398 nm was detected. C18-DHS and C18-PHS were reacted with the AQC reagent and employed as standards for quantification.

### ISP-1 uptake assay

Yeast cells were cultured in YPD medium to the logarithmic growth phase, treated with 500 ng mL^−1^ ISP-1 for 1.5 h, and then harvested and washed with water. Yeast cells were suspended in 0.1 M KCl, and the cell suspension was vortexed six times for 30 sec with a half-volume of glass beads, and then the mixture was adjusted to a final acetic acid concentration of 0.1 M. ISP-1 in the lysate was extracted by successive addition and mixing of reagents as follows: (1) 2.5 volumes of methanol and 1.25 volumes of chloroform with 10 min of shaking; and (2) 1.25 volumes of chloroform and 1.25 volumes of water with overnight shaking at 4°C. The phases were separated by centrifugation, and the organic phase was recovered, dried, and resuspended in 60 mM triethylamine/methanol. HPLC analysis was performed using a C18 column (4.6 × 250 mm, XDB-C18; Hewlett-Packard) on a Shimadzu LC10A series liquid chromatography system using gradient elution with a total flow rate of 1.0 mL min^−1^. Solution A contained 1.73 g of CH_3_COONa-3H_2_O, 0.55 mL of phosphoric acid and 0.09 mL of triethylamine per liter, and Solution B consisted of 60% acetonitrile. The timeline for gradient elution was as follows: 0–20 min, Solution A, 90–67%; 20–50 min, Solution A, 67–0%; and 50–70 min, Solution A, 0%. Lipid-reacted AQC reagent was detected as in the LCB measurements. ISP-1 reacted with the AQC reagent (AQC-ISP-1) was eluted at 44.8 min. This peak was further evaluated by MALDI-TOF-MS analysis, whereby the expected sizes ([M+H]^+^ = 572.2 m/z; [M+Na]^+^ = 594.1 m/z) were confirmed. Therefore, this peak was employed as the standard for quantification. The concentration of ISP-1 was normalized to the OD_600_ value of each strain.

### Western blotting

Whole-cell extracts were prepared from logarithmically growing cells. Yeast cells were harvested and resuspended in lysis buffer (50 mM Tris-HCl, pH 7.5, 0.5 mM EGTA, 1.5 mM MgCl_2_, 1 mM PMSF, protease inhibitor cocktail [Nacalai Tesque, Kyoto, Japan], 5 mM dithiothreitol, 25 mM *β*-glycerophosphate, 50 mM NaF, 0.5% Triton X-100). To lyse yeast cells, the cell suspension was vortexed with a half-volume of glass beads, as described previously (Momoi et al. 2004). Unbroken cells and debris were removed by centrifugation at 800*g* for 5 min, and the supernatants were treated with SDS-PAGE (sodium dodecyl sulfate polyacrylamide gel electrophoresis) sample buffer and boiled for 5 min for denaturation. Protein concentrations were determined using a Bio-Rad protein assay kit (Hercules, CA). In standard SDS-PAGE (7.5% acrylamide) and Western blotting, 30 *μ*g of protein per lane for Ypk1 and Pgk1 were loaded on the gels. The endogenous levels of Ypk1, Fpk1, Lcb4, and Bap2 were detected using a polyclonal antibody specific for each protein (Iwaki et al. 2005; Tanoue et al. 2005). GST-Ypk1 and GST-Fpk1/2 were visualized using rabbit antibodies directed against GST (Upstate Biotechnology). A chemiluminescent substrate, Chemilumi-One (Nacalai Tesque), and LAS-4000 Mini (Fujifilm, Tokyo, Japan) were used for signal detection. To quantify protein abundance, we measured the signal intensities of the bands, and the signals were normalized relative to that of Pgk1 as a loading control using the ImageGage software (Fujifilm).

### In vitro kinase assay

Cells that overexpressed GST-Fpk1 or/and GST-Fpk2 under the control of the *ADH1* promoter were lysed, and the GST-fusion proteins were precipitated by glutathione-Sepharose 4B (GE Healthcare, Little Chalfont, UK). GST-fusion proteins isolated from total lysates (400 *μ*g protein) of yeast cells were used in the reaction. Recombinant GST-Ypk1 (WT or the 71A mutant) was expressed in *Escherichia coli*, isolated using a column of glutathione-Sepharose 4B and eluted with glutathione, as reported previously (Tanoue et al. 2005). GST-Ypk1 (20 *μ*g) was used in the reaction, unless otherwise stated. The kinase reaction (100 *μ*L) was performed in kinase buffer (25 mM Tris-HCl, pH 7.5, 5 mM *β*-glycerophosphate, 2 mM DTT, 0.1 mM Na_3_VO_4_, 10 mM MgCl_2_). The reaction was started by addition of 10 *μ*L of a solution containing 50 *μ*M cold ATP, 3 *μ*Ci [*γ*-^32^P]-ATP, and 10 mM MgCl_2_, incubated for 30 min at 30°C with tapping at 3-min intervals, and terminated with the addition of 5× SDS-PAGE sample buffer. The samples were heated to 98°C for 5 min. The proteins were separated by SDS-PAGE, and the gel was subsequently dried. Phosphorylated bands were detected by autoradiography using BAS-2500 (Fujifilm). Proteins separated by SDS-PAGE were also subjected to Western blotting using anti-GST antibodies.

### Determination of the phosphorylation site on Ypk1 by liquid chromatography–tandem mass spectrometry

Yeast cells harboring *P*_*GAL1*_*-GST-YPK1* were grown to log phase in SR medium, and GST-Ypk1 expression was induced by addition of 4% galactose. PHS (20 *μ*M) was added to the medium, and the cells were cultured for 15 min before lysate preparation. The GST-fusion proteins were bound to glutathione-Sepharose 4B resin and subjected to SDS-PAGE. A retarded band corresponding to phosphorylated Ypk1 was isolated from the Coomassie brilliant blue (CBB)-stained gel. The band was subjected to in-gel digestion with 12.5 ng mL^−1^ trypsin. The resulting mixture was analyzed by LTQ (Thermo Electron, Waltham, MS) liquid chromatography-tandem mass spectrometry (LC-MS/MS), and the corresponding proteins were searched using the Mascot software (Matrix Science, London, UK) (Hachiro et al. 2013), which is used to identify proteins by matching mass spectroscopic data with information from the National Center for Biotechnology Information (NCBI; http://www.ncbi.nlm.nih.gov) and Swiss-Prot (http://us.expasy.org) protein databases.

### Determination of duramycin resistance

Overnight-cultured yeast cells were diluted to an OD_600_ of 0.1 and then cultured at 30°C in preheated SD selective medium for 1 h. For the spot assay, cells (2 *μ*L) were spotted in 10-fold serial dilutions on YPD plates that contained duramycin (5 *μ*g mL^−1^). The plates were then incubated at 30°C for 1.5 days. For the halo assay, overnight-cultured yeast cells (∼2 × 10^8^) were plated on YPD plates, and duramycin (1 mM) was then dropped onto the plates. The plates were incubated for 3 days. Results of duramycin resistance are shown in accordance with a previous report to facilitate comparison (Roelants et al. 2010). However, resistance was not reproducible regardless of the assay (spot or halo) used.

### DNA microarray

Auxotroph-matched BY4741*-HIS3* control cells and *fpk1/2Δ* cells were cultured to log phase, and mRNA was enriched using a combination of the hot phenol method and an mTRAP mRNA Isolation kit (Active Motif, Carlsbad, CA). The DNA microarray analysis was carried out as reported previously (Koike et al. 2004), except that a yeast cDNA microarray (Yeast Chip ver. 2-14; DNA Chip Research Inc., Yokohama, Japan) was used. The experiments adhered to the MIAME guidelines (Brazma et al. 2001), and the obtained data were submitted to the GEO database (http://www.ncbi.nlm.nih.gov/geo/) under submission number GSE42083. Extracted genes with expression levels that exhibited more than twofold difference between samples are listed in Table S2.

### Metabolic labeling of de novo sphingolipid biosynthesis

The sphingolipid profile of the *fpk1/2Δ* strain was examined by de novo labeling with [^3^H]-serine, as reported previously (Sun et al. 2000). Briefly, log-phase cells were labeled with [^3^H]-serine for 4 h. The sphingolipid fraction was enriched and resolved by thin layer chromatography (TLC). The radioactivity in each band was visualized using a BAS2500 and tritium-imaging plates (Fujifilm).

## Results and Discussion

### Deletion-mediated ISP-1 resistance (DIR)

**Figure 1:**
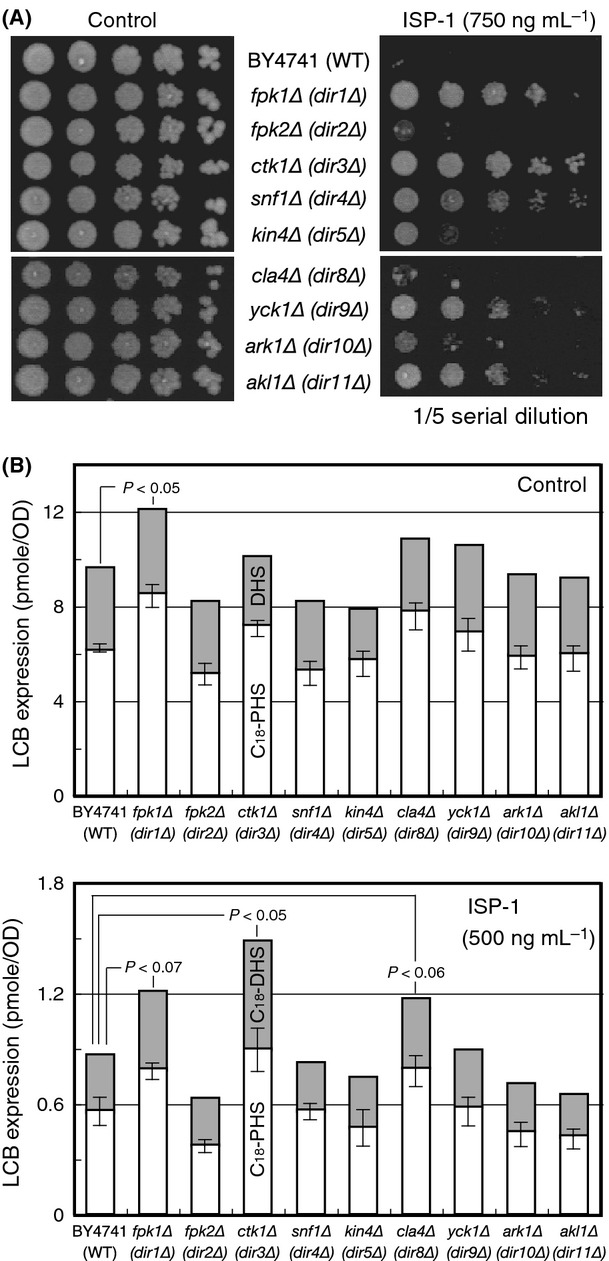
Identification of *DIR* genes. (A) ISP-1 resistance profiling of the *DIR* genes. Kinase-knockout (*DIR*) cells and control BY4741 (WT) cells were assessed for ISP-1 resistance on YPD-ISP-1 (750 ng mL^−1^) plates. The yeast culture was diluted serially as indicated and incubated at 30°C for 3 days. Plate images were acquired using the Printgraph camera system (ATTO, Tokyo, Japan). (B) LCB profiles of *DIR* cells. The LCB profiles of the *DIR* strains were determined in HPLC-based assays, as described in Experimental Procedures. The cellular levels of C18-PHS (white box) and C18-DHS (gray box) are expressed as incremented columns for each strain cultured in YPD medium (top). The LCB levels after 3 h of treatment with 500 ng mL^−1^ ISP-1 are also plotted (bottom). Each value shown is the mean of three independent experiments, and the standard deviation of error (SDE) for each experiment is indicated as a bar. Statistically significance differences compared to the WT control were evaluated using Student's *t* test and are shown when *P* < 0.1.

ISP-1/myriocin treatment causes acute cellular sphingolipid depletion, as ISP-1 is a potent inhibitor of serine palmitoyltransferase, which is responsible for the first step in sphingolipid biosynthesis (Miyake et al. 1995). Mammalian and yeast cells that are deprived of sphingolipids undergo cell death upon ISP-1 treatment. Reversal of this lethal phenotype can be exploited to screen for genes that are involved in sphingolipid-mediated signal transduction. Characterization of such kinase(s) will facilitate an understanding of protein kinase signaling integration that mediates generation of cellular sphingolipids, information about which is limited at present. We carried out an assay in which many protein kinases and several lipid kinase-knockout cells were systematically assessed for ISP-1 resistance. We spotted 105 commercially available kinase-knockout strains on ISP-1 plates and compared their resistance to that of the WT control strain on the same plate. Nine *DIR* genes were isolated by screening using serial dilution spotting on plates. This method ensures that cells are treated at different densities to examine ISP-1 resistance (Fig. 1A). We also observed a similar resistance profile of these *DIR* mutants in a liquid culture system (data not shown).

We expected that genetic screening of chemical compounds would identify genes that modulate cell membrane integrity. In the case of *DIR* screening, we selected genes resistant to ISP-1. In fact, some of the *DIR* genes had deletion phenotypes that indicated sensitivity to stress conditions, listed in the *Saccharomyces* Genome Database (http://www.yeastgenome.org/). In contrast, *DIR* strains acquired resistance to ISP-1 despite a general membrane-weakening phenotype, indicating that membrane integrity has only a limited effect on the ISP-1-resistant phenotype of these *DIR* strains (Table 1). We also identified knockout strains sensitive to ISP-1 (*d*eletion-mediated *I*SP-1 *v*ulnerability or DIV) (Table S1). We believe that loss of membrane integrity (Levin 2005) could account for the ISP-sensitivity of some *DIV* strains. However, loss of membrane integrity is not the sole cause of changes in ISP-1 sensitivity; *DIV1/YPK1/SLI2* are also directly associated with cellular sphingolipids. The complete results of ISP-1 resistance screening are presented in Table S1. Genome-wide chemical biology screening utilizing an array of knockout cells has previously been reported (Hillenmeyer et al. 2008). These genome-wide knockout strains (including protein kinases) were treated with the compounds (including ISP-1/myriocin A) for cellular fitness. When their fitness defect values were compared to the ISP-1 resistance strength found in the present study, these two assays generally agreed and the overall Pearson's correlation coefficient was positive (*r* > 0.36). Specifically, *DIV* genes and their fitness defective genes showed stronger agreement than *DIR* genes and gained fitness genes. As the ISP-1 resistance assay was used to measure growth differences, essential genes cannot be examined; therefore, more sophisticated assays are required to examine the entire kinome. Moreover, the presence of redundancy between similar kinases makes it difficult to detect potential links between sphingolipids. Probably for this reason, our screening assay did not identify *PKH1/2* as *DIV* genes, although Pkh1/2 was shown to be involved in sphingolipid-mediated signaling event(s) (Friant et al. 2001; Tanoue et al. 2005; Luo et al. 2008; Berchtold et al. 2012).

**Table 1:** List of *DIR* genes.

DIR number	Gene name	Kinase family	Description
*DIR1*	*FPK1*	AGC, S6K	Flippase regulation
*DIR2*	*KIN82/FPK2*	AGC, S6K	Flippase regulation
*DIR3*	*CTK1*	CMGC	C-terminal domain kinase for RNA pol. II
*DIR4*	*SNF1*	CAMK	AMPK for glucose starvation signal
*DIR5*	*KIN4*	CAMK	Inhibits the mitotic exit network, MEN
*DIR8*	*CLA4*	STE	PAK (p21-activated kinase) family
*DIR9*	*YCK1*	CK1	PM casein kinase I isoform
*DIR10*	*ARK1*	ARK/PRK	Required for endocytosis
*DIR11*	*AKL1*	ARK/PRK	Member of the Ark kinase family

### LCB levels in DIR mutants

We observed that nine of 105 protein kinase mutations resulted in acquired ISP-1 resistance, but the mode of resistance for these kinase mutants remained unclear. Therefore, we first attempted to type *DIR* mutants. Although ISP-1 depletes multiple molecular species of sphingolipids, recent progress in the field indicates that loss of LCBs, rather than downstream complex sphingolipid species, causes lethality (Epstein et al. 2012). We thus determined basal LCB levels in the *DIR* mutants using fluorescence HPLC. The *fpk1Δ/dir1* cells exhibited a statistically significant increase in basal LCB level (Fig. 1B). Thus, Fpk1 kinase signals attenuated basal LCB levels. In contrast, other *DIR* strains did not have significantly elevated basal LCB levels, showing that increased basal LCB expression is not a common mechanism of ISP-1 resistance in these strains.

We next explored the effects of ISP-1 treatment on cellular LCB levels. When compared to the control strain, strains with deletions of *fpk1Δ/dir1*,*ctk1Δ/dir3* (Lee and Greenleaf 1991), and *cla4Δ*/*dir8* (Cvrckova et al. 1995) retained higher levels of LCBs 3 h after ISP-1 treatment (Fig. 1B), the time point at which ISP-1 starts to affect yeast viability (Sun et al. 2000). The weak ISP-1 resistance, despite the marked restoration of LCB levels in *cla4Δ* cells, could be caused by the “slow growth” phenotype of this strain. Strains with deletions of *fpk2Δ/dir2*,*snf1Δ/dir*4, *kin4Δ/dir5*,*yck1Δ/dir9*,*ark1Δ/dir10*, and *akl1Δ/dir11* did not show significantly altered levels of LCBs, although there was a tendency toward stronger basal LCB levels in *yck1Δ/dir9* cells. Among these mutants, *fpk2Δ/dir2*,*kin4Δ/dir5*, and *ark1Δ/dir10* exhibited very limited resistance, as compared with the WT control. Thus, it is difficult to determine whether LCB levels are associated with the resistance phenotype. Given the stronger resistance levels of *snf1Δ/dir4* (Carlson et al. 1981) and *akl1Δ/dir11* (Takahashi et al. 2006), these kinases may be involved in signaling events downstream of the sphingolipids. Overall, Dir protein kinases were dispersed in the AGC, CAMK, CMGC, CK, and STE families within the yeast kinome, although the AGC protein kinase family was mainly studied in the context of cellular sphingolipids.

The ISP-1 resistance of *DIR* mutants was not uniform. Therefore, resistance could be mediated via several independent mechanisms. It has been reported that aureobasidin A (AbA), an inhibitor of complex sphingolipid biosynthesis (Fig. S1A), causes yeast cell death, perhaps due to accumulation of ceramide species with C26 acyl chains (Nagiec et al. 1997; Epstein et al. 2012). The AbA resistance spectra of *DIR* mutants were different from that of ISP-1 in that resistance was notably weaker in the *fpk1Δ/dir1*,*yck1Δ/dir9,* and *akl1Δ/dir11* strains (Fig. S1B). Therefore, these kinases could have a more direct relationship with LCB species than ceramides or complex sphingolipids. The similar resistance of the *ctk1Δ/dir3* and *snf1Δ/dir4* strains to ISP-1 and AbA further confirms that these protein kinases have a distinct mode of resistance to *fpk1Δ/dir1* cells. The LCB-level typing of *DIR* strains revealed that ISP-1/aureobasidin A resistance is achieved by various independent mechanisms; thus, more detailed analysis is required. Notably, the *fpk1Δ/dir1* strain showed elevated LCB levels regardless of ISP-1 treatment, as well as the strongest resistance among *DIR* strains.

### Basic characterization of Fpk1/Dir1 and Fpk2/Dir2

**Figure 2:**
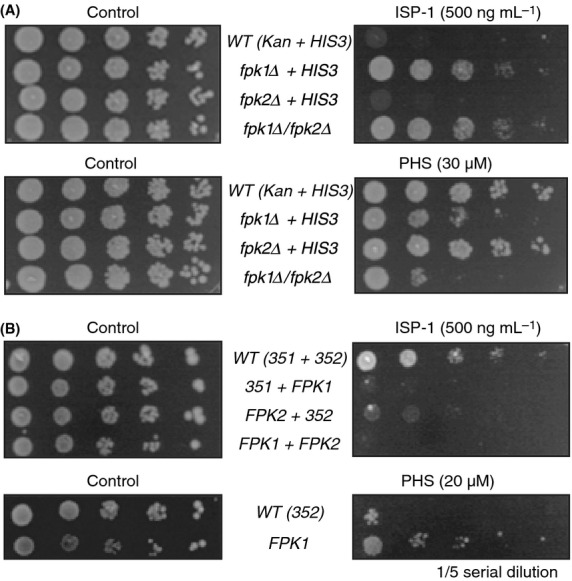
Fpk1/Dir1 and Fpk2/Dir2 in ISP-1 resistance assays. (A) Effect of double deletion. Single-and double-deletion strains of *FPK1/2* were adjusted for genetic background by the mean of *HIS3* and/or *KanMx* expression from a single-copy vector as indicated. Auxotrophy-matched cells were assessed for resistance to 750 ng mL^−1^ ISP-1 or 30 *μ*M PHS on SD plates. The data shown are representative of several independent experiments. (B) Effect of multicopy *FPK1/2* expression. Single or double expression of *FPK1/2* through the use of YEp352/1 multicopy vectors was assessed in resistance assays, as described in (A). Auxotrophy of these strains was matched with empty vector transformation when necessary (351 and/or 352).

Among the *DIR* genes, *FPK1* and *FPK2* encode a homologous set of protein kinases that may play redundant roles. We evaluated these genes because both paralogs were positive in the screening. Indeed, it has been reported that Fpk1/2 kinases have redundant roles in aminophospholipid flippase regulation (Nakano et al. 2008), and mutation of *FPK1* causes ISP-1 resistance (Roelants et al. 2010). Thus, Fpk1/2 kinases are likely associated with cellular lipids. The *fpk1/2Δ* cells show potent ISP-1 resistance, similar to *fpk1Δ* cells (Fig. 2A). Unlike our screening assays (Fig. 1A), the resistance of *fpk2Δ* was not apparent in SD medium. In accordance with the deletion phenotype, overexpression of Fpk1 sensitized yeast cells to ISP-1 (Fig. 2B), although a kinase-inactive mutant did not exhibit the same effect (data not shown). Thus, it was evident that *FPK1* gene dosage was negatively correlated with the strength of ISP-1 resistance. Although LCBs are essential for yeast viability, supplementation with excess PHS, a major LCB species in yeast, is cytotoxic (Skrzypek et al. 1998; Chung et al. 2001). We also examined the resistance of these cells to PHS to understand its relationship with LCBs. Fpk1 abundance was positively correlated with resistance to PHS, indicating that cellular LCB levels are responsible for the phenotype (Fig. 2A and B). Here, expression of *FPK2* exhibited an additive effect. Taken together, these data suggest that *FPK1/2* expression enhanced the effect of PHS but reduced that of ISP-1; both results are consistent with the elevated LCB levels of *fpk1Δ* cells.

The ISP-1 resistance profile of *FPK1* was the opposite of that of *YPK1/SLI2*;*YPK1* overexpression and deletion caused ISP-1 resistance and hypersensitivity, respectively. However, overexpression of Ypk1 did not result in sensitization to PHS-mediated growth arrest (Y. Yamane-Sando and H. Takematsu, unpubl. results). It was previously proposed that Ypk1 could phosphorylate Fpk1, and Fpk1 could phosphorylate Ypk1 (Roelants et al. 2010). However, the relationships of the Fpk1–Ypk1 signaling axis to cellular LCB levels and resistance to ISP-1/PHS remain unclear, and further investigations are required. Therefore, we re-examined the Fpk1/2-Ypk1 association to characterize the link between these kinases and LCBs.

### Fpk1/2 kinases are responsible for Ypk1 phosphorylation in a sphingolipid-dependent manner

Ypk1 is a multicopy suppressor of ISP-1-mediated cell lethality, and Ypk1 phosphorylation could be visualized as a band-shift in normal SDS-PAGE assays after PHS treatment (Fig. S2A) (Sun et al. 2000). After PHS treatment, the slow-migrating GST-Ypk1 was excised from the gel and subjected to trypsinization. By LC-MS/MS analysis, the only phosphorylated polypeptide fragment identified had the sequence KGTINPSNSSVVPVRVSYDASSSTSTVR, corresponding to amino acids 55–82 of Ypk1, and serine residue 71 (Ser71, underlined) was phosphorylated. Indeed, this site was one of two candidate phosphorylation sites proposed in a previous alanine mutation study (Roelants et al. 2010). Similar to the effect of ISP-1 treatment, knock-in mutation of Ser71 to alanine (Ypk1^S71A^) suppressed the mobility shift observed upon PHS treatment, although Ser51 was also proposed as a phosphorylation site (Fig. S2B). On the basis of these results, we concluded that Ser71 is the phosphorylation site responsible for the observed PHS-mediated major mobility shift. In contrast to previous Ypk1 overexpression experiments (Roelants et al. 2010), we found that a single-deletion mutation of either *fpk1Δ* or *fpk2Δ* did not attenuate Ypk1 phosphorylation, whereas double-deletion mutation resulted in loss of the PHS-mediated major shift (Fig. S2C). We noted that a minor shift was detectable under this latter condition, indicating that Ypk1 could be phosphorylated by another protein kinase(s) in a PHS-dependent manner.

When Fpk1 was visualized by Western blotting using antiserum raised against the N-terminal portion of the protein, it appeared as a very broad band, indicating that Fpk1 itself is a phosphorylated protein (Fig. S2D). One of the candidate protein kinases is Ypk1. However, overexpression of Ypk1 did not affect the smear pattern of Fpk1 phosphorylation (data not shown). In any case, Fpk1 exhibited a stronger phenotype than Fpk2 in all assays. Therefore, of these two kinases, Fpk1 plays the more prominent role. On the basis of these results, we subsequently focused on Fpk1.

### Direct phosphorylation of Ypk1 by Fpk1 in vitro

**Figure 3:**
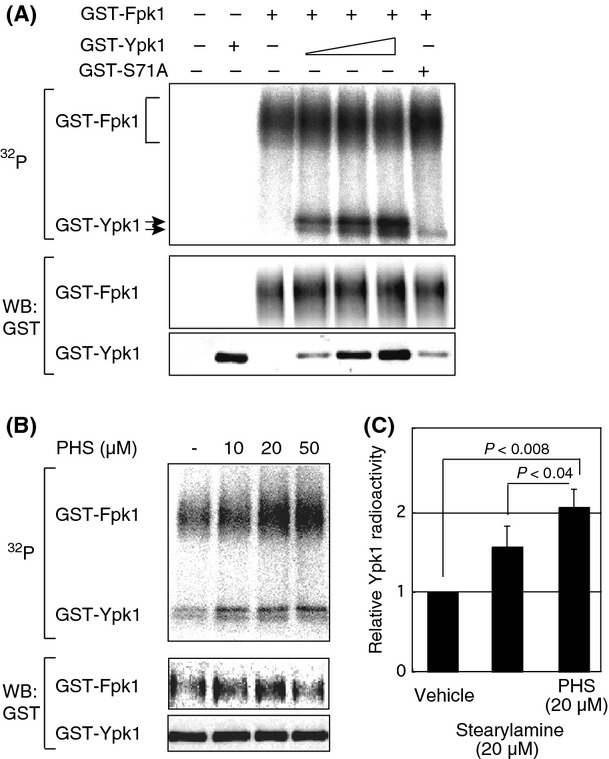
In vitro assay of Fpk1 kinase activity toward Ypk1. (A) The in vitro kinase assay for Fpk1 utilized recombinant Ypk1 as the substrate. GST-Fpk1 purified from yeast cells was incubated with a graded concentration of Ypk1 or the S71A mutant and visualized by autoradiography after SDS-PAGE. Fpk1 and Ypk1 used in the reaction were also visualized by Western blotting. Fpk1-mediated phosphorylation of Ypk1 resulted in a major mobility-shifted band. (B) Effect of PHS on Fpk1 kinase activity toward Ypk1. Fpk1 activity was measured in the presence of the indicated concentration of PHS to examine the effect of LCB on Fpk1 activity. (C) Evaluation of the effect of PHS. To evaluate PHS-mediated activation of Fpk1, incorporation of radioactivity into Ypk1 was measured in three independent experiments. The relative mean radioactivity levels in the presence of PHS and stearylamine are shown in the columns. The SDE values of the experiments are indicated as bars on top of the columns. Student's *t* test was used to determine *P*-values.

We next examined whether Fpk1 could directly phosphorylate Ypk1 at Ser71. An in vitro kinase assay using GST-Fpk1 immunoprecipitated from yeast cells without substrate resulted in a broad band of radioactive GST-Fpk1. Thus, GST-Fpk1 isolated from yeast cells has autophosphorylation activity (Fig. 3A). When recombinant Ypk1 was added as a substrate, similar to a previous study (Roelants et al. 2010), phosphorylation of Ypk1 by Fpk1 was detected. Ypk1 phosphorylation by Fpk1 in vitro resulted in the same mobility shift observed in cells treated with PHS (Fig. 3A). This indicated that sphingolipid-mediated phosphorylation was triggered by Fpk1. We also used the S71A mutant of Ypk1 as the substrate to determine the significance of the site. Ypk1^S71A^ phosphorylation in vitro was severely attenuated, and the resultant shifted band was diminished (Fig. 3A). These data indicated that Ser71 was the major site of Fpk1-mediated phosphorylation, and phosphorylation by Fpk1 was sufficient for the observed band-shift. As we observed a faint signal with the S71A substrate, other less preferred phosphorylation sites could exist in Ypk1. Potential phosphorylation site(s) include Ser51, as suggested previously (Roelants et al. 2010).

We next determined the effect of PHS on Fpk1 activity toward Ypk1, because the shifted band was induced in PHS-treated cells (Fig. S2A). As expected, Fpk1 activity toward Ypk1 was stimulated by PHS (Fig. 3B). In terms of the dose-dependency of the effect, the signal appeared saturated at 20 *μ*M (Fig. 3B). Consistently, autophosphorylation of Fpk1 was also increased under these conditions, indicating that PHS can directly activate Fpk1 regardless of the substrate (Fig. 3B). Similar to Ypk1 activity toward Lsp1 or Pil1 (Zhang et al. 2004), a control amino lipid, stearylamine, also resulted in subtle activation. However, Fpk1 activity in the presence of PHS was stronger than under control conditions, which appeared to be of biological significance (Fig. 3C) as PHS is the physiological lipid present in cells. We also noted that the faint, unshifted band was grossly unaffected by PHS (Fig. 3B). Taken together, our results indicated that Fpk1 is an LCB-activated kinase that preferentially phosphorylates Ser71 of Ypk1, resulting in a major mobility shift on SDS-PAGE, although other minor phosphorylation site(s) may be present.

### Neither ISP-1 resistance nor duramycin sensitivity was altered by Ypk1-S71A mutation

**Figure 4:**
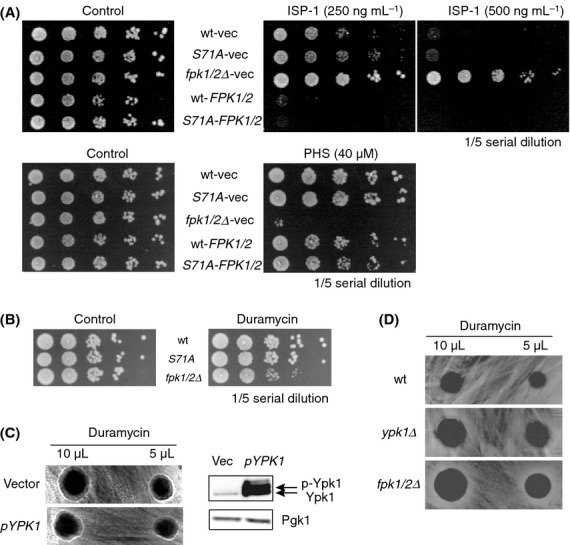
Impact of Ser71 phosphorylation of Ypk1 in ISP-1 and duramycin resistance assays. (A) Resistance profile of *ypk1*^*S71A*^ knock-in cells. *ypk1*^*S71A*^ knock-in cells that harbored the indicated vectors were assessed for resistance to ISP-1 and PHS. Vec indicates empty vector transformants, used to normalize auxotrophy. (B) Duramycin resistance was measured using spot assays. Although loss of *FPK1/2* resulted in the sensitization of cells to duramycin, the knock-in strain of the *ypk1*^*S71A*^ mutant was similar to the WT control. (C) Duramycin sensitivity was measured in halo assays, and was indicated by the diameter of the halo around a drop of duramycin of the indicated concentration. Halos were not altered in cells that overexpressed Ypk1 (pYPK1, left). Western blotting evaluation of Ypk1 overexpression in these strains (right). (D) Duramycin sensitivity of *ypk1Δ* cells. Duramycin sensitivity was evaluated by measuring the diameter of the halo around a drop of duramycin in auxotroph-matched WT, *ypk1Δ*, and *fpk1/2Δ* cells. Duramycin sensitivity was elevated in both *ypk1Δ* and *fpk1/2Δ* cells.

To understand the role of the Fpk1–Ypk1 signaling axis in ISP-1 resistance, we first evaluated the impact of Ser71 phosphorylation on Fpk1 function. The prediction was that Fpk1 negatively controls Ypk1-mediated ISP-1 resistance. However, the *S71A* knock-in mutation did not affect ISP-1 resistance in either WT or Fpk1/2-overexpressing cells (Fig. 4A). Consistently, the PHS sensitization phenotype was not altered by S71A mutation of Ypk1 (Fig. 4A) These observations indicated that Ypk1 phosphorylation by Fpk1 is not involved in the PHS/ISP-1 resistance phenotype. Therefore, we evaluated other Fpk1 downstream events. The aminophospholipid flippase Dnf1/2 has been reported to be regulated by Fpk1/2-mediated phosphorylation (Nakano et al. 2008). As duramycin is a cyclic tetrapeptide targeted toward cell-surface phosphatidylethanolamine (PE), *fpk1/2Δ* cells were sensitive to duramycin, likely because PE is more abundant on the surface of cells of this strain (Nakano et al. 2008). We examined duramycin resistance to examine the involvement of Ypk1 in relation to Fpk1/2 function. Although Fpk1 phosphorylated Ypk1 at Ser71, the *ypk1*^*S71A*^ knock-in cells did not show altered sensitivity to duramycin (Fig. 4B); thus Fpk1-mediated phosphorylation of Ypk1 is not involved in the duramycin resistance. In contrast to previous findings (Roelants et al. 2010), we did not observe a loss-of-resistance phenotype in cells that overexpressed Ypk1, even when a multicopy vector was used to drive Ypk1 expression, a condition under which Ypk1 abundance were greatly enhanced (Fig. 4C). In addition, *YPK1* deletion did not confer duramycin resistance as reported; rather, deletion resulted in increased sensitivity to duramycin compared to control cells (Fig. 4D). Taken together, our data did not support Fpk1-mediated Ypk1 suppression or Ypk1-mediated Fpk1 suppression in the context of resistance toward PHS, ISP-1, or duramycin under our study conditions. To clarify the difference, we employed the same resistance assay to that used in the previous study (Roelants et al. 2010), and used strains with similar backgrounds. Therefore, differences in the strains used do not explain the discrepant results. Our duramycin resistance assay seemed reliable because *fpk1/2Δ* cells were consistently sensitive to duramycin (Fig. 4B) (Nakano et al. 2008). These data may reflect the combination of the “slow growth and/or compromised cell wall” phenotype of *ypk1Δ* cells (Chen et al. 1993) and the flippase defect of *fpk1/2Δ* cells (Nakano et al. 2008). To support this notion, *fpk1/2Δ* cells, but not *ypk1*^*S71A*^ mutant cells, were resistant to ISP-1 and sensitive to PHS (Fig. 4A). In conclusion, a functional relationship between Fpk1-mediated Ser71 phosphorylation of Ypk1 and cell phenotype (susceptibility profiles to PHS, ISP-1, and duramycin) was not established, although Fpk1/2 kinases do phosphorylate, Ypk1 and Fpk1 activity was enhanced by the presence of PHS. Therefore, further explanation of the ISP-1-resistant and PHS-sensitive phenotype of *fpk1/2Δ* cells is necessary.

### ISP-1 uptake is regulated by Fpk1/2

**Figure 5:**
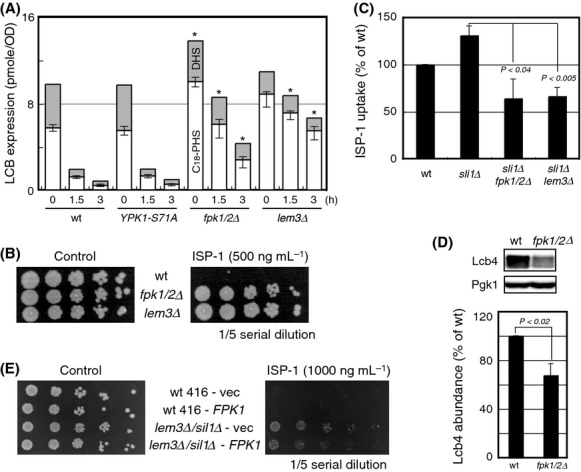
LCB levels are increased in the *fpk1/2Δ* mutant. (A) LCB levels in *fpk1/2Δ* cells. LCB levels were measured in the indicated mutant strains before and after ISP-1 treatment in YPD medium as in Figure 1B. LCB levels were measured by HPLC, and the values in the columns indicate the mean levels of C18-PHS and C18-DHS with SDE values from three independent experiments. *P < 0.01, compared to the WT control for each condition, as evaluated using Student's *t* test. (B) ISP-1 resistance assay for *lem3Δ* cells. The ISP-1 resistance assay was carried out as described in Figure 1A using auxotrophy-matched WT,*fpk1/2Δ*, and *lem3Δ* strains. (C) ISP-1 uptake by *fpk1/2Δ* or *lem3Δ* cells in the *sli1Δ* background. The indicated cells were treated with 500 ng mL^−1^ ISP-1 for 1.5 h, and ISP-1 uptake was measured by fluorescence HPLC, as described in Experimental Procedures. The relative mean ISP-1 levels in three independent experiments are plotted. The statistical significance of differences was evaluated using Student's *t* test. (D) Lcb4 suppression in *fpk1/2Δ* cells. Lysates were prepared from cells in log phase, and Western blotting was carried out as described in Figure 3C. The membrane was probed with antiserum against Lcb4 or Pgk1 (left). The graph on the right shows the relative abundance of Lcb4 from three independent experiments. The statistical significance of differences was evaluated using Student's *t* test. (E) Resistance of flippase-deficient cells could be affected by *FPK1* overexpression. Control or *lem3Δ/sli1Δ* cells that harbored the indicated vectors were assessed for resistance to ISP-1. pRS416 empty vector transformants were used to normalize auxotrophy. Vec indicates the empty vector for *FPK1* overexpression.

If Fpk1/2 is not a negative regulator of Ypk1, some alternative pathway must confer ISP-1 resistance upon *fpk1/2* mutation (Fig. 2A). Evaluation of LCB levels in these cells may lead to the formulation of an alternative hypothesis. Dnf1/2 is an aminophospholipid flippase phosphorylated by Fpk1/2, and plasma membrane localization of Dnf1/2 is defective in *lem3Δ* cells (Fig. S3A) (Hanson et al. 2003). Here, we compared the WT control with *ypk1*^*S71A*^ knock-in, *fpk1/2Δ*, and *lem3Δ* cells. In the absence of ISP-1, LCB levels were identical in WT and *ypk1*^*S71A*^ cells. PHS accumulated in *fpk1/2Δ* cells (Fig. 5A) to a greater extent than in cells with the single *fpk1Δ* mutation (Fig. 1B). Interestingly, the PHS/dihydrosphingosine (DHS) ratio was skewed in *lem3Δ* cells without a statistically significant alteration in the overall LCB level (Fig. 5A). In conclusion, basal LCB levels cannot be attributed to flippase regulation by Fpk1/2. In contrast to WT and *ypk1*^*S71A*^ cells, both *fpk1/2Δ* cells and *lem3Δ* cells were resistant to LCB depletion upon ISP-1 treatment (Fig. 5A). This was in agreement with the ISP-1 resistance of triple flippase mutant cells (Roelants et al. 2010). As *lem3Δ* cells also exhibited ISP-1 resistance (Fig. 5B), we examined whether ISP-1 uptake is regulated by the aminophospholipid flippase. Fpk1/2 has been reported to negatively regulate flippase action and induce the inward movement of aminolipids (Nakano et al. 2008). It is noteworthy that Dnf1/2 can mobilize phosphatidylcholine, phosphatidylethanolamine, and phosphatidylserine; this represents a rather loose specificity for a flippase. ISP-1 consists of an amino group and a carboxyl group with a hydrophobic stretch (Fig. S3B), although this structure more closely resembles that of LCBs than that of phospholipids. Nevertheless, we measured direct ISP-1 uptake from the culture medium by *fpk1/2Δ* cells using fluorescence HPLC. *SLI1* encodes an ISP-1 *N*-acetyltransferase that acetylates the amino group of the molecule to inactivate its SPT inhibitor activity (Momoi et al. 2004). As we utilized an amino group-labeling method to detect cellular ISP-1, we first assessed the ISP-1 content of ISP-1-treated WT and *sli1Δ* cells. As expected, ISP-1 (with the amino group) levels were higher in *sli1Δ* cells (Fig. 5C), indicating that ongoing acetylation by Sli1 obscures ISP-1 detection in the WT strain in our uptake assay. Therefore, we utilized the *sli1Δ* background to accurately measure ISP-1 uptake. Both *lem3Δ* and *fpk1/2Δ* cells showed attenuated uptake of ISP-1 (Fig. 5C). Therefore, we concluded that Fpk1/2 signals Dnf1/2 to induce ISP-1 uptake, thus altering cellular resistance to ISP-1. Although our assay does not discriminate whether flippase components are directly involved in ISP-1 uptake or whether disruption of phospholipid asymmetry in the plasma membrane alters subsequent ISP-1 uptake by modulating cell wall integrity (Pomorski et al. 2003) or other membrane feature(s). However, it is clear that regulation of ISP-1 uptake results in the resistance of *fpk1/2Δ*. Another important question was how *fpk1/2Δ* cells increase basal LCB levels in the absence of ISP-1 (Fig. 5A) and increase sensitivity to PHS (Fig. 4A), both of which are ISP-1-independent phenotypes. We hypothesize that these kinases also negatively regulate basal LCB levels.

### Transcriptome analysis of fpk1/2Δ cells

To elucidate the function(s) of Fpk1/2, we compared the transcriptomes of WT and *fpk1/2Δ* cells using DNA microarrays. The *fpk1/2Δ* cells suppressed gene expression of the functionally major type of sphingosine kinase, *LCB4*, and the sphingosine-1-phosphate lyase, *DPL1* (Table 2). As both of these genes encode enzymes involved in the LCB degradation pathway (Fig. S4A), simultaneous repression of these two genes is expected to result in the intracellular accumulation of nonphosphorylated forms of LCBs. In this respect, it has been reported that *lcb4/dpl1Δ* cells lack detectable levels of LCB phosphates and that LCB levels are more than 10-fold higher in *lcb4/dpl1/ysr2Δ* cells (Kim et al. 2000). Moreover, single deletion of *LCB4* was shown to result in a *ca*. fourfold increase in LCBs (Ferguson-Yankey et al. 2002) (Sano et al. 2005). This indicates that phosphorylation of LCBs by Lcb4 is the rate-limiting step in the LCB degradation pathway (Iwaki et al. 2007). Therefore, cooperative downregulation of degradation pathway enzymes could explain the increase in LCB levels and in PHS sensitivity in *fpk1/2Δ* cells. Consistent with the transcriptional changes, the abundance of Lcb4 was attenuated as determined by Western blotting (Fig. 5D). Under these conditions, phosphorylation of Lcb4 remained unchanged, in agreement with a previous report that Pho85 is the protein kinase responsible for Lcb4 phosphorylation (Iwaki et al. 2005). Dnf1/2 flippase function is dispensable for Lcb4 expression regulation as Lcb4 abundance was not altered in the *lem3Δ* strain (Fig. S4B), in which total LCB levels were unchanged but showed an increased ratio of PHS to DHS (Fig. 5A). To determine whether minor differences in LCBs in *fpk1/2Δ* could alter the ISP-1-resistant phenotype, we examined loss of ISP-1 resistance in Fpk1-overexpressing strains utilizing the *lem3Δ/sli1Δ* strain as a genetic background. This strain was used to eliminate the functions of flippase; that is, ISP-1 uptake (*lem3Δ*) and ISP-1 inactivation (*sli1Δ*). Indeed, *pFPK1*-mediated loss of resistance was detected in the flippase-deficient strain (Fig. 5E). Therefore, relatively minor changes in LCBs can cause phenotypic changes in the resistance assay. On the basis of these results, we concluded that transcriptional regulation of the LCB degradation pathway is affected by a signaling event downstream of Fpk1/2. Currently, the transcriptional factor(s) involved in regulating this transcriptomic change downstream of Fpk1/2, thereby enhancing LCB degradation, remain(s) to be identified. It is interesting to speculate that the Fpk1/2 kinases function to sense LCB levels, as Fpk1/2 activities seem to be modulated in vitro by the addition of PHS (Fig. 3B). Identification of such an Fpk1/2 substrate could provide an understanding of LCB homeostasis.

**Table 2:** Transcriptomic analysis of *fpk1/2Δ* cells.

Systemic name	Gene name	Description	Fold-expression (fpk1Δfpk2Δ/wt)
YBR068C	*BAP2*	High-affinity leucine permease	3.51
YDR072C	*IPT1*	M(IP)2C synthase	2.67
YKL004W	*AUR1*	IPC synthase	2.49
YCR034W	*FEN1/ELO2*	Fatty acid elongase	2.48
YPL057C	*SUR1*	MIPC synthase subunit	2.02
YOR171C	*LCB4*	Sphingoid long-chain base kinase	0.24
YDR477W	*SNF1/DIR4*	AMPK catalytic subunit	0.39
YDR208W	*MSS4/SLI6*	PhosIns 4-P 5 kinase	0.40
YIL105C	*SLM1*	Synthetically lethal with *mss4*	0.45
YDR294C	*DPL1*	Dihydrosphingosine phosphate lyase	0.48

Genes that showed a ≥ twofold increase or decrease in expression in the *fpk1/2Δ* cells were identified by competitive two-color hybridization assay using the cDNA microarray. Sorted genes of interest are listed here; a full array-wide list of genes is presented in Table S2. Each gene is listed with the ORF name, gene name, brief description, and fold-difference in expression, as compared to the control.

Microarray experiments also showed that the genes encoding enzymes involved in complex-type sphingolipids, that is, *CSG1*,*IPT1*,*AUR1,* and *ELO2*, were upregulated (Fig. S4A), whereas the expression levels of the genes encoding the ceramide synthase subunits were not altered. Irrespective of the putative upregulation of these enzymes, de novo biosynthesis of complex-type sphingolipids was not significantly altered in *fpk1/2Δ* cells (Fig. S4C). In accordance with these findings, de novo biosynthesis of sphingolipids was more strongly affected by incorporation of serine into the pathway than actual SPT activity (Cowart and Hannun 2007). Alternatively, complex-type sphingolipids and LCBs may be regulated independently, albeit not exclusively. In agreement with this notion, the ceramide band was somewhat weaker in only *fpk1/2Δ* cells in de novo labeling experiments with radioactive serine (Fig. S4C).

In addition to the sphingolipid-related enzyme genes, our microarray comparison also detected induction of the *BAP2* gene, which encodes a branched amino acid permease. Western blotting revealed that Bap2 abundance was increased in *fpk1/2Δ* cells (Fig. S4D). Thus, Fpk1/2 may control cellular uptake of branched amino acids. Another notable finding in the microarray results was that *fpk1/2Δ* cells repressed *SNF1/DIR4*, Snf1 substrate, and many ribosome-related genes (Table S2) (Breeden and Nasmyth 1987). Therefore, signaling downstream of Fpk1/2 may involve complex intracellular events, such as ribogenesis or nutrient uptake, which may be affected by the availability of glucose or amino acids. Our DIR screening suggested that Snf1, the yeast homolog of the AMP-activated protein kinase family, could be involved in sphingolipid-mediated signaling pathways. Indeed, recent systems biological approaches utilizing gene ontology annotations suggested a potential relationship between Snf1 and cellular sphingolipids, and showed that deletion of the *SNF1* gene could cause ISP-1 resistance (Yucel and Ulgen 2013). This report listed 15 other genes – including two *DIV* and three *DIR* genes (including *SNF1*) – possibly involved in the context of cellular sphingolipids. Although *SNF1* was the only gene exceeding the twofold threshold in *fpk11/2Δ* DNA microarray analyses, the expression of *SCS7* and *CSG2* (among the 16 genes listed) was also increased by roughly 1.5-fold in *fpk1/2Δ* cells (data not shown). Therefore, some of the listed genes seem to be related to cellular sphingolipids regulated by Fpk1/2.

### Functional assessment of a mammalian kinase of the S6K/RSK family

**Figure 6:**
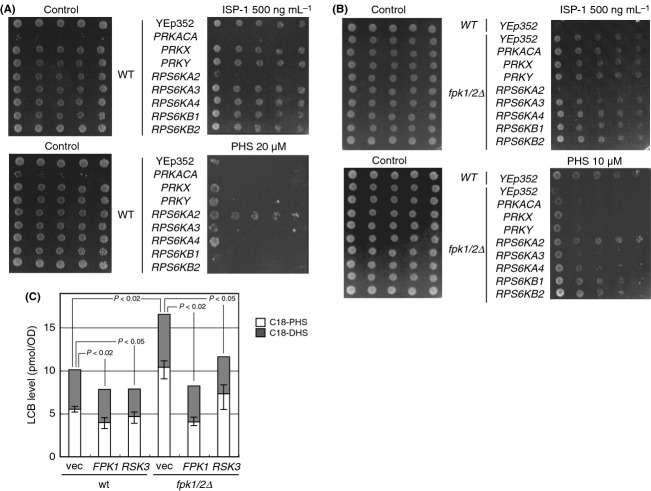
Assessment of a mammalian counterpart of *FPK1/2*. ISP-1 resistance of human cDNA-expressing WT (A) and *fpk1/2Δ* (B) cells. The assay for resistance to ISP-1 or PHS was carried out as described in Figure 2A. Human protein kinase cDNA was expressed using a multicopy YEp352 vector harboring the promoter and terminator of the *ADH1* gene. (C) LCB level of *RPS6KA2/RSK3*-expressing cells. The basal levels of LCBs in the indicated cells everexpressing Fpk1 or RSK3 cultured in SD medium (to retain the plasmid) were quantified as shown in Figure 1B.

Fpk1 and Fpk2 belong to the AGC kinase family and exhibit sequence similarities to S6K/RSK family protein kinases (Fig. S5A). Although Fpk1/2 proteins align with p60-S6 kinases (S6K), a functional counterpart of S6K, Sch9, has been reported in yeast (Urban et al. 2007). The transcriptomic data indicated that one of the events downstream of Fpk1/2 could involve the biosynthesis of ribosomes (Table S2). In any case, Fpk1/2 kinases could be functionally conserved in mammals. Therefore, functional assays were used to characterize relationships between human and yeast genes (Fig. S5A). As intracellular Fpk1/2 functionality could be measured using the ISP-1 resistance assay in yeast cells, we evaluated eight protein kinases of mammalian origin (Fig. S5B). When expressed in *fpk1/2Δ* cells, only *RPS6KA2/p90-RSK3* (*RSK3*) rescued the *fpk1/2Δ* phenotype with respect to both ISP-1 and PHS (Fig. 6A and B) despite PRKX and PRKY exhibiting greater sequence similarity. As expected from the lack of a resistance phenotype of the *ypk1*^*S71A*^ mutant, *RSK3* expression did not rescue the Ypk1 Ser71 phosphorylation defect of *fpk1/2Δ* (Fig. S5C). These results further indicate that the Fpk1–Ypk1 phosphorylation axis is distinct from the ISP-1 resistance activity of *fpk1/2Δ* cells. Currently, the physiological significance of the Fpk1–Ypk1 branch remains to be clarified, although it is clear that Fpk1 does not negatively regulate Ypk1. Identification of the specific substrate(s) affected by the *ypk1*^*S71A*^ knock-in mutation may reveal its significance, as point mutations could affect specific downstream events (Tanoue et al. 2005). Our results indicate that mammalian cells retain a functionally equivalent kinase in their kinome in the *RSK3* of the p90-S6K/RSK family, which was previously suggested to function downstream of MAP kinase signaling (Bignone et al. 2007). To determine the Fpk1 function (LCB degradation or flippase regulation) that is complemented by *RSK3*, we examined the LCB profiles of these cells in the absence of ISP-1. *RSK3* expression suppressed LCB levels that were increased by *fpk1/2* deletion (Fig. 6C). We found that RSK3 is likely the functional counterpart to Fpk1, regulating LCB degradation rather than ISP-1 uptake to confer ISP-1 resistance (Fig. S6A).

## Concluding Remarks

In this study, we isolated *DIR* genes to gain insight into kinome functionality with regard to sphingolipid metabolism/signaling. We show here for the first time that the Fpk1/2 (Dir1/2) proteins have dual functions in terms of regulating ISP-1 uptake and LCB degradation (Fig. 5). This seems to explain the robust ISP-1 resistance of *fpk1/2Δ* cells (Fig. S6A). The function of Fpk1/2 is not limited to cell survival in response to compound treatment. Rather, Fpk1/2 also functions to maintain cellular LCB levels and enable signaling (Fig. S6B), which appeared to be evolutionarily conserved.

## Supporting Information

**Figure S1.**Proliferation of *DIR*strains after inhibition of sphingolipid biosynthesis. (A) Sphingolipid biosynthetic pathways in yeast. The biosynthetic pathway of sphingoli-pids is shown schematically. Inhibitors used in this studyare shown in blue. Molecular species of the sphingoid long-chain bases (LCBs) and complex sphingolipids are indicated on the left. (B) Aureobasidin A resistance of *DIR*strains based on the results of spot assays. Spot assays were performed as described in Figure 1A, except that a YPD-aureo-basidin A (AbA, 40 ng mL^−1^) plate was used. **Figure S2.**Sphingolipid-mediated phosphorylation of Ypk1 is mediated by the Fpk1/2 kinases. (A) Purification of PHS-treated Ypk1. Recombinant Ypk1 conjugated to a GST-tag was purified from yeast cells that harbored pEG (KG), and the protein was resolved by SDS-PAGE and stained with Coomassie brilliant blue. The mobility-shifted bands (arrowheads) were excised from the gel and subjected to MALDI-TOF/MS-MS analysis to identify phosphorylation sites. (B) Evaluation of PHS-induced Ser71 phosphoryla-tion. A lysate of nontagged endogenous/knock-in Ypk1 was assessed by Western blotting after PHS or ISP-1 treatment. Upon PHS treatment, WT Ypk1 accumulated the phosphor-ylated species, whereas the Ypk1S71A mutant did not. (C) Effect of Fpk1/2 expression on sphingolipid-induced phosphorylation of endogenous Ypk1. Cells that carried a single deletion of *FPK1*or *FPK2*or a double mutation were compared with WT cells. Auxotrophy of each strain was matched with *KanMx/HIS3*vector transformation. Cells treated with PHS or ISP-1 were evaluated. Fpk1 and Ypk1 were visualized using specific antisera. Pgk1 was employed as a control. The arrowhead indicates the minor shift of the Ypk1 band in *fpk1/2Δ*cells. (D) Basal phosphorylation of Fpk1. Cell lysates were treated with calf intestinal phosphatase (CIP) and evaluated by Western blotting. The mobility of the Fpkl band increased upon phosphatase digestion, indicating that Fpk1 is a phosphoprotein. **Figure S3.**Flippase regulation by Fpk1/2. (A) Effects of Lem3 on flippase expression: Dnf1/2 is complexed with Lem3 in the endoplasmic reticulum to sort Dnf1/2 to the plasma membrane. Therefore, *lem3Δ*cells fail to express active Dnf1/2 complex on the plasma membrane. (B) Structures of the flippase Dnf1/2 substrates PC, PS, PE, and ISP-1. **Figure S4.**Transcriptomic analyses 1 of the *fpkl/2Δ*cells. (A) Biosynthetic pathway for sphingolipids in budding yeast. The species of sphingolipids are connected with arrows that represent the enzymatic reactions responsible. Colored arrows represent downregulated (blue) or upregu-lated (red) enzyme genes in *fpk1/2Δ*cells, as compared with the WT cells based on the results of cDNA microarray analysis. Full microarray data have been submitted to the GEO database under submission number GSE42083. (B) Examination of the role of flippase in Lcb4 expression. Lcb4 abundance was determined based on Western blotting experiments, as in Figure 5D. Lcb4 signal in flippase-defi-cient *lem3Δ*cells was compared with WT control (left). Band intensities were calculated from three independent experiments and are shown as mean values of differences relative to control cells (right). Bars in each graph represent SDE among experiments. WT and *lem3Δ*expressed similar levels of Lcb4, indicating that flippase regulation of Fpk1/2 is dispensable for Lcb4 expression. (C) Assessment of de novo biosynthesis of complex-type sphingolipids. Cells of each genotype were metabolically labeled with [^3^H]-serine with ISP-1. The lipid fractions were isolated and separated using thin layer chromatography. Radioactivity was detected in the BAS-2500 using a tritium plate. Sphingolipid species are indicated. (D) Analysis of Bap2 expression. *BAP2*encodes a branched amino acid permease. Bap2 expression was monitored in the cells of the indicated genotypes using Western blotting. Pgk1 was utilized as a loading control. **Figure S5.**Assessment of a mammalian functional counterpart of *FPK1/2.*(A) Sequence similarities among the indicated AGC kinases from humans and budding yeasts are expressed in a phylogenetic tree using ClustalW software.(B) ISP-1 resistance of human cDNA-expressing WT cells. The assay for resistance to ISP-1 or PHS was performed as described in Figure 2A, except that the WT was used. Human protein kinase cDNA was expressed using a multicopy vector with the *ADH1*promoter. (C) PHS-mediated Ypk1 phosphorylation in human kinase cDNA-expressing cells. Western blotting was performed as shown in Figure S2B. Recovery of Ser71 phosphorylation (causing slow migration) of Ypk1 was assessed with PHS. None of the human kinases recovered Ser71 phosphorylation in the *fpk1/2Δ*background. This result indicated that Ser71 phos-phorylation is independent of ISP-1 resistance. **Figure S6.**Schematic presentation of Fpk1/2 function in relation 1 to cellular LCBs. (A) Putative role of Fpk1/2 in LCB suppression Fpk1/2 regulates the phosphorylation of Dnf1/2 to control ISP-1 uptake. Fpk1/2 also regulated *LCB4*expression to control LCB degradation. A combination of these two events is responsible for the robust deletion-mediated ISP-1 resistance of *fpk1/2Δ*cells. In addition, Fpk1/2 control phosphorylation of Ypk1 at Ser71, although downstream events remain unclear. (B) Possible homeo-static balancing of cellular LCB levels. Previous studies showed that Orm1/2 control cellular LCB levels by interacting with SPT. Orm1/2 can be phosphorylated by Ypk1, which thus could control SPT activity. Pkh1/2-mediated phosphorylation of the activation loop on Ypk1 is important for Ypk1 activity, and Pkh1/2 activity could be stimulated in an LCB-dependent manner. A combination of these mechanisms could form a positive-feedback loop rather than negative-feedback to maintain LCB levels, which would play a role in induction. Fpk1/2 is also an AGC kinase, which requires activation-loop phosphoryla-tion. Therefore, Pkh1/2 could be involved in the Fpk1/2 branch. Our results suggested that Fpk1/2 could be stimulated by LCBs and suppress the degradation pathway of LCBs. This pathway is likely involved in homeostatic balancing of LCB levels. **Table S1.**Screening for *DIR/DIV* genes. **Table S2.** Transcriptome analysis of *fpk1/2Δ* cells. **Table S3.** Genotypes of the yeast strains used in this study. **Table S4.** Plasmids used in this study.
