# Supplementary material for: Fpk1/2 kinases regulate cellular sphingoid long-chain base abundance and alter cellular resistance to LCB elevation or depletion
Source: Microbiologyopen. 2014 Feb 10;3(2):196–212. doi: 10.1002/mbo3.160 (PMC3996568; doi:10.1002/mbo3.160)
Supplement: Supplementary file 1 — Figure S1.Proliferation of DIRstrains after inhibition of sphingolipid biosynthesis. (A) Sphingolipid biosynthetic pathways in yeast. The biosynthetic pathway of sphingoli-pids is shown schematically. Inhibitors used in this studyare shown in blue. Molecular species of the sphingoid long-chain bases (LCBs) and complex sphingolipids are indicated on the left. (B) Aureobasidin A resistance of DIRstrains based on the results of spot assays. Spot assays were performed as described in Figure 1A, except that a YPD-aureo-basidin A (AbA, 40 ng mL−1) plate was used. Figure S2.Sphingolipid-mediated phosphorylation of Ypk1 is mediated by the Fpk1/2 kinases. (A) Purification of PHS-treated Ypk1. Recombinant Ypk1 conjugated to a GST-tag was purified from yeast cells that harbored pEG (KG), and the protein was resolved by SDS-PAGE and stained with Coomassie brilliant blue. The mobility-shifted bands (arrowheads) were excised from the gel and subjected to MALDI-TOF/MS-MS analysis to identify phosphorylation sites. (B) Evaluation of PHS-induced Ser71 phosphoryla-tion. A lysate of nontagged endogenous/knock-in Ypk1 was assessed by Western blotting after PHS or ISP-1 treatment. Upon PHS treatment, WT Ypk1 accumulated the phosphor-ylated species, whereas the Ypk1S71A mutant did not. (C) Effect of Fpk1/2 expression on sphingolipid-induced phosphorylation of endogenous Ypk1. Cells that carried a single deletion of FPK1or FPK2or a double mutation were compared with WT cells. Auxotrophy of each strain was matched with KanMx/HIS3vector transformation. Cells treated with PHS or ISP-1 were evaluated. Fpk1 and Ypk1 were visualized using specific antisera. Pgk1 was employed as a control. The arrowhead indicates the minor shift of the Ypk1 band in fpk1/2Δcells. (D) Basal phosphorylation of Fpk1. Cell lysates were treated with calf intestinal phosphatase (CIP) and evaluated by Western blotting. The mobility of the Fpkl band increased upon phosphatase digestion, indicating that Fpk1 is a phosphop [file mbo30003-0196-sd1.pdf]

Fpk1/2 kinases regulate cellular sphingoid long-chain base (LCB) abundance and alter cellular resistance to LCB elevation or depletion

SUPPLEMENTAL FIGURE LEGENDS

Supplemental Figure S1: Proliferation of *DIR* strains after inhibition of sphingolipid biosynthesis

A. Sphingolipid biosynthetic pathways in yeast.

The biosynthetic pathway of sphingolipids is shown schematically. Inhibitors used in this study are shown in blue. Molecular species of the sphingoid long chain bases (LCBs) and complex sphingolipids are indicated on the left.

B. Aureobasidin A resistance of *DIR* strains based on the results of spot assays

Spot assays were performed as described in Figure 1A, except that a YPD-aureobasidin A (AbA, 40 ng ml^{-1}) plate was used.

Supplemental Figure S2: Sphingolipid-mediated phosphorylation of Ypk1 is mediated by the Fpk1/2 kinases.

A. Purification of PHS-treated Ypk1.

Recombinant Ypk1 conjugated to a GST-tag was purified from yeast cells that harboured pEG (KG), and the protein was resolved by SDS-PAGE and stained with Coomassie brilliant blue. The mobility-shifted bands (arrowheads) were excised from the gel and subjected to MALDI-TOFMS-MS analysis to identify phosphorylation sites.

B. Evaluation of PHS-induced Ser71 phosphorylation.

A lysate of non-tagged endogenous/knock-in Ypk1 was assessed by Western blotting after PHS or ISP-1 treatment. Upon PHS treatment, WT Ypk1 accumulated the phosphorylated species, whereas the $Ypk1^{S71A}$ mutant did not.

C. Effect of Fpk1/2 expression on sphingolipid-induced phosphorylation of endogenous Ypk1.

Cells that carried a single deletion of *FPK1* or *FPK2* or a double mutation were compared with WT cells. Auxotrophy of each strain was matched with *KanMx/HIS3* vector transformation. Cells treated with PHS or ISP-1 were evaluated. Fpk1 and Ypk1 were visualized using specific antisera. Pgk1 was employed as a control. The arrowhead indicates the minor shift of the Ypk1 band in *fpk1/2*Δ cells.

D. Basal phosphorylation of Fpk1.

Cell lysates were treated with calf intestinal phosphatase (CIP) and evaluated by Western blotting. The mobility of the Fpk1 band increased upon phosphatase digestion, indicating that Fpk1 is a phosphoprotein.

Supplemental Figure S3: Flippase regulation by Fpk1/2.

A. Effects of Lem3 on flippase expression: Dnf1/2 is complexed with Lem3 in the endoplasmic reticulum to sort Dnf1/2 to the plasma membrane. Therefore, *lem3*Δ cells fail to express active Dnf1/2 complex on the plasma membrane.

B. Structures of the flippase Dnf1/2 substrates PC, PS, PE and ISP-1.

Supplemental Figure S4: Transcriptomic analyses of the *fpk1/2*Δ cells.

A. Biosynthetic pathway for sphingolipids in budding yeast. The species of sphingolipids are connected with arrows that represent the enzymatic reactions responsible. Coloured arrows represent down-regulated (blue) or up-regulated (red) enzyme genes in *fpk1/2*Δ cells, as compared with the WT cells based on the results of cDNA microarray analysis. Full microarray data have been submitted to the GEO database under submission number GSE42083.

B. Examination of the role of flippase in Lcb4 expression.

Lcb4 abundance was determined based on Western blotting experiments, as in Figure 5D. Lcb4 signal in flippase-deficient *lem3*Δ cells was compared with WT control (left). Band intensities were calculated from three independent experiments and are shown as mean values of differences relative to control cells (right). Bars in each graph represent SDE among experiments. WT and *lem3*Δ expressed similar levels of Lcb4, indicating that flippase regulation of Fpk1/2 is dispensable for Lcb4 expression.

C. Assessment of *de novo* biosynthesis of complex-type sphingolipids.

Cells of each genotype were metabolically labelled with [^{3}H]-serine with ISP-1. The lipid fractions were isolated and separated using thin layer chromatography. Radioactivity was detected in the BAS-2500 using a tritium plate. Sphingolipid species are indicated.

D. Analysis of Bap2 expression.

BAP2 encodes a branched amino acid permease. Bap2 expression was monitored in the cells of the indicated genotypes using Western blotting. Pgk1 was utilized as a loading control.

Supplemental Figure S5: Assessment of a mammalian functional counterpart of *FPK1/2*.

A. Sequence similarities among the indicated AGC kinases from humans and budding yeasts are expressed in a phylogenetic tree using ClustalW software.

B. ISP-1 resistance of human cDNA-expressing WT cells.

The assay for resistance to ISP-1 or PHS was performed as described in Figure 2A, except that the WT was used. Human protein kinase cDNA was expressed using a multicopy vector with the *ADH1* promoter.

C. PHS-mediated Ypk1 phosphorylation in human kinase cDNA-expressing cells.

Western blotting was performed as shown in Supplemental Figure S2B. Recovery of Ser71 phosphorylation (causing slow migration) of Ypk1 was assessed with PHS. None of the human kinases recovered Ser71 phosphorylation in the *fpk1/2*Δ background. This result indicated that Ser71 phosphorylation is independent of ISP-1 resistance.

Supplemental Figure S6: Schematic presentation of Fpk1/2 function in relation to cellular LCBs

A. Putative role of Fpk1/2 in LCB suppression

Fpk1/2 regulates the phosphorylation of Dnf1/2 to control ISP-1 uptake. Fpk1/2 also regulated *LCB4* expression to control LCB degradation. A combination of these two events is responsible for the robust deletion-mediated ISP-1 resistance of *fpk1/2*Δ cells. In addition, Fpk1/2 control phosphorylation of Ypk1 at Ser71, although downstream events remain unclear.

B. Possible homeostatic balancing of cellular LCB levels.

Previous studies showed that Orm1/2 control cellular LCB levels by interacting with SPT. Orm1/2 can be phosphorylated by Ypk1, which thus could control SPT activity. Pkh1/2-mediated phosphorylation of the activation loop on Ypk1 is important for Ypk1 activity, and Pkh1/2 activity could be stimulated in an LCB-dependent manner. A combination of these mechanisms could form a positive-feedback loop rather than negative-feedback to maintain LCB levels, which would play a role in induction. Fpk1/2 is also an AGC kinase, which requires activation-loop phosphorylation. Therefore, Pkh1/2 could be involved in the Fpk1/2 branch. Our results suggested that Fpk1/2 could be stimulated by LCBs and suppress the degradation pathway of LCBs. This pathway is likely involved in homeostatic balancing of LCB levels.

SUPPLEMENTAL TABLES

Supplemental Table S1

Screening for *DIR/DIV* genes

Supplemental Table S2

Transcriptome analysis of *fpk1/2*Δ cells

Extracted list of cDNA microarray experiments comparing the transcriptomes of the auxotroph-matched WT cells and *fpk1/2*Δ cells. Data was expressed as fold-expression compared to control (WT).

Supplemental Table S3

Genotypes of the yeast strains used in this study.

	Genotype	Source/Ref.
BY4741	*MATa his3-1 leu2-0 met15-0 ura3-0*	(Brachmann et al., 1998)
dir1Δ (fpk1Δ)	BY4741 *fpk1::KanMX4*	This study*
dir2Δ (fpk2Δ)	BY4741 *fpk2::KanMX4*	(Giaever et al., 2002)
dir3Δ (ctk1Δ)	BY4741 *ctk1::KanMX4*	(Giaever et al., 2002)
dir4Δ (snf1Δ)	BY4741 *snf1::KanMX4*	(Giaever et al., 2002)
dir5Δ (kin4Δ)	BY4741 *kin4:KanMX4*	(Giaever et al., 2002)
dir8Δ (cla4Δ)	BY4741 *cla4::KanMX4*	(Giaever et al., 2002)
dir9Δ (yck1Δ)	BY4741 yck1*::KanMX4*	(Giaever et al., 2002)
dir10Δ (ark1Δ)	BY4741 *ark1::KanMX4*	(Giaever et al., 2002)
dir11Δ (akl1Δ)	BY4741 *akl1::KanMX4*	(Giaever et al., 2002)
fpk1Δfpk2Δ	BY4741 *fpk1::KanMX4 fpk2::HISMX6*	This study
YPK1^{S71A}	BY4741 *ypk1*::*ypk1*^{S71A}*-HISMX6*	This study
ypk1Δ	BY4741 *ypk1::HISMX6*	(Shimobayashi *et al.*, 2010)**
lem3Δ	BY4741 *lem3::KanMX4*	(Giaever et al., 2002)
sli1Δ	BY4741 *sli1::URA3*	This study
fpk1Δfpk2Δsli1Δ	BY4741 *fpk1::KanMX4 fpk2::HISMX6 sli1::URA3*	This study
lem3Δsli1Δ	BY4741 *lem3::KanMX4 sli1::URA3*	This study

* The *fpk1Δ* strain originally obtained from the knockout collection showed a *fpk1Δ* genotype appropriate for a knockout strain. However, we also noted that this strain showed a heat stress-sensitive phenotype, which was not rescued by *FPK1* supplementation. Therefore, other mutations were also present in this strain. Therefore, we constructed a fresh *fpk1Δ* mutant strain in this study.

** Based on the results of PCR-genotyping, the *ypk1Δ* strain obtained from the knockout collection harbours both knockout (appropriate sized band was amplified by knockout project primer) and unknown alleles of *YPK1*. Moreover, we detected the Ypk1 band on Western blotting in this *ypk1Δ* strain, indicating that this is not a true deletion mutant. This was further supported by the observation that commercial strains grew normally, whereas recreated *ypk1Δ* strains showed slow growth phenotypes, as expected from a previous report. We used recreated *ypk1Δ* strains in our previous studies and in this study.

Supplemental Table S4

Plasmids used in this study.

Plasmid name	Gene	Description
351 (YEp351)	Control	*LEU2* marker in multicopy vector
352 (YEp352)	Control	*URA3* marker in multicopy vector
HIS3 (pRS413)	Control	*HIS3* marker in single-copy vector
pRS416	Control	*URA3* marker in single-copy vector, for PCR-based homologous recombination
pFA6a-HIS3MX6	Control	For PCR-based homologous recombination
FPK1 (FPK1/Yep351)	*FPK1*	Multicopy vector with own promoter *FPK1*
FPK1 (FPK1/Yep352)	*FPK1*	Multicopy vector with own promoter *FPK1*
FPK2 (FPK2/Yep351)	*FPK2*	Multicopy vector with own promoter *FPK2*
ADH1-FPK1/Yep352	*FPK1*	Multicopy vector with *ADH1* promoter *FPK1*
ADH1-FPK2/Yep351	*FPK2*	Multicopy vector with *ADH1* promoter *FPK2*
pYPK1/YEp351	*YPK1*	(Sun et al., 2000)
pYPK1^{S71A}*/YEp351*	*YPK1*^{S71A}	*pYPK1* with S71A mutation
YPK1^{S71A}*/pFA6a-HIS3MX6*	*YPK1*^{S71A}	For PCR-based homologous recombination
P_{GAL1}*-GST-YPK1*	*YPK1*	(Zhu et al., 2000)
P_{GAL1}*-GST-YPK1*^{S71A}	*YPK1*^{S71A}	PCR-based homologous recombination P_{GAL1}*-GST-YPK1*
YPK1/pGEX4T-1	*YPK1*	*E. coli* expression vector with GST tag and *YPK1*
YPK1^{S71A}*/pGEX4T-1*	*YPK1*^{S71A}	*E. coli* expression vector with GST tag and *YPK1*^{S71A}
BAP2(N-term)/pGEX4T-1	*BAP2(N)*	*E. coli* expression vector with GST tag and Bap2 N-term
FPK1(N-term)/pGEX4T-1	*FPK1(N)*	*E. coli* expression vector with GST tag and Fpk1 N-term
PRKACA/YEp352	*PRKACA*	Multicopy vector with *ADH1* promoter *PRKACA*
PRKX/YEp352	*PRKX*	Multicopy vector with *ADH1* promoter *PRKX*
PRKY/YEp352	*PRKY*	Multicopy vector with *ADH1* promoter *PRKY*
PRS6KA2 (RSK3)/YEp352	*PRS6KA2*	Multicopy vector with *ADH1* promoter *PRS6KA2*
PRS6KA3/YEp352	*PRS6KA3*	Multicopy vector with *ADH1* promoter *PRS6KA3*
RPS6KA4/YEp352	*RPS6KA4*	Multicopy vector with *ADH1* promoter *RPS6KA4*
RPS6KB1/YEp352	*RPS6KB1*	Multicopy vector with *ADH1* promoter *RPS6KB1*
RPS6KB2/YEp352	*RPS6KB2*	Multicopy vector with *ADH1* promoter *RPS6KB2*

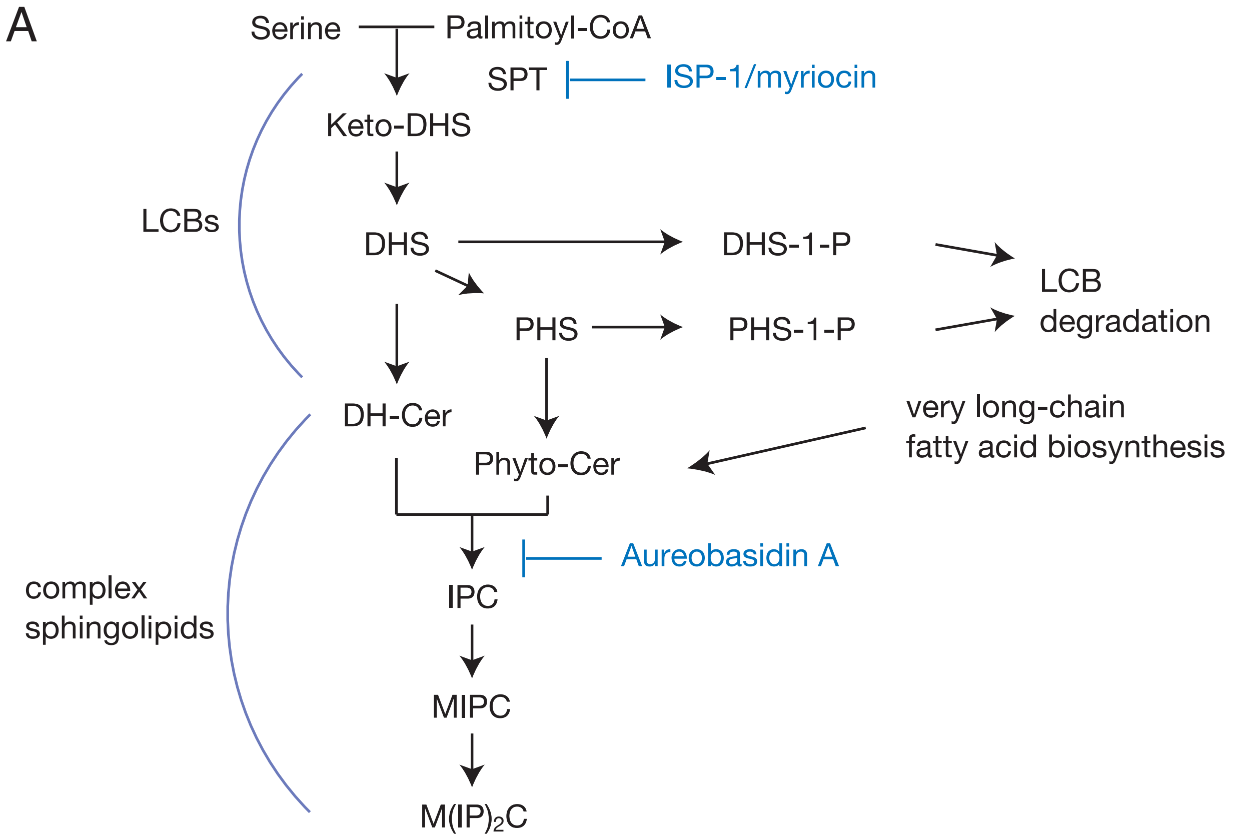
A
Serine
Palmitoyl-CoA
SPT
ISP-1/myriocin
Keto-DHS
LCBs
DHS
DHS-1-P
LCB
degradation
PHS
PHS-1-P
DH-Cer
Phyto-Cer
very long-chain
fatty acid biosynthesis
Aureobasidin A
complex
sphingolipids
IPC
MIPC
M(IP)2C

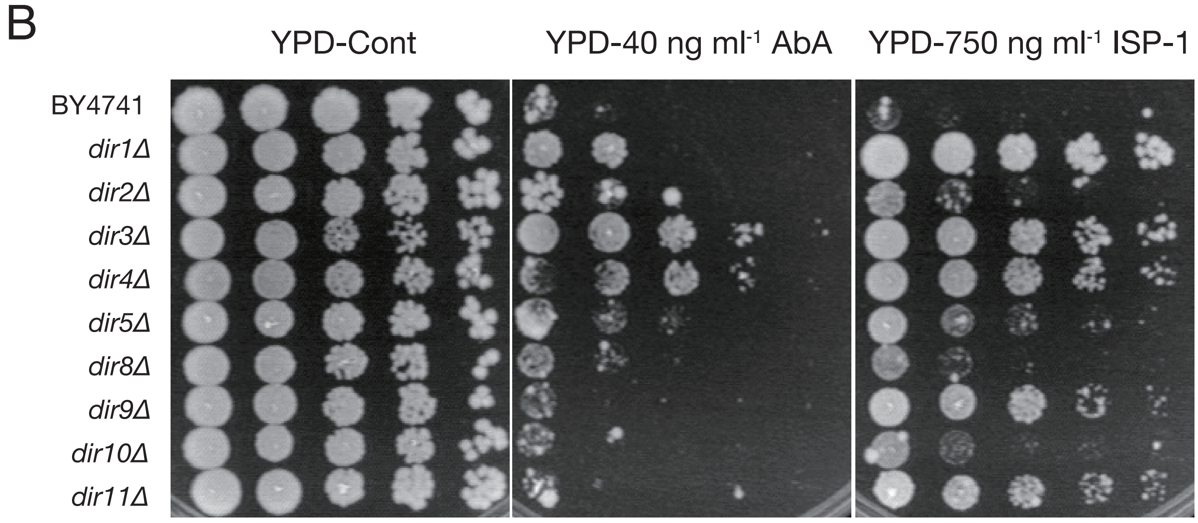
B
YPD-Cont
YPD-40 ng ml-1 AbA
YPD-750 ng ml-1 ISP-1
BY4741
dir1Δ
dir2Δ
dir3Δ
dir4Δ
dir5Δ
dir8Δ
dir9Δ
dir10Δ
dir11Δ

Supplemental Figure S2

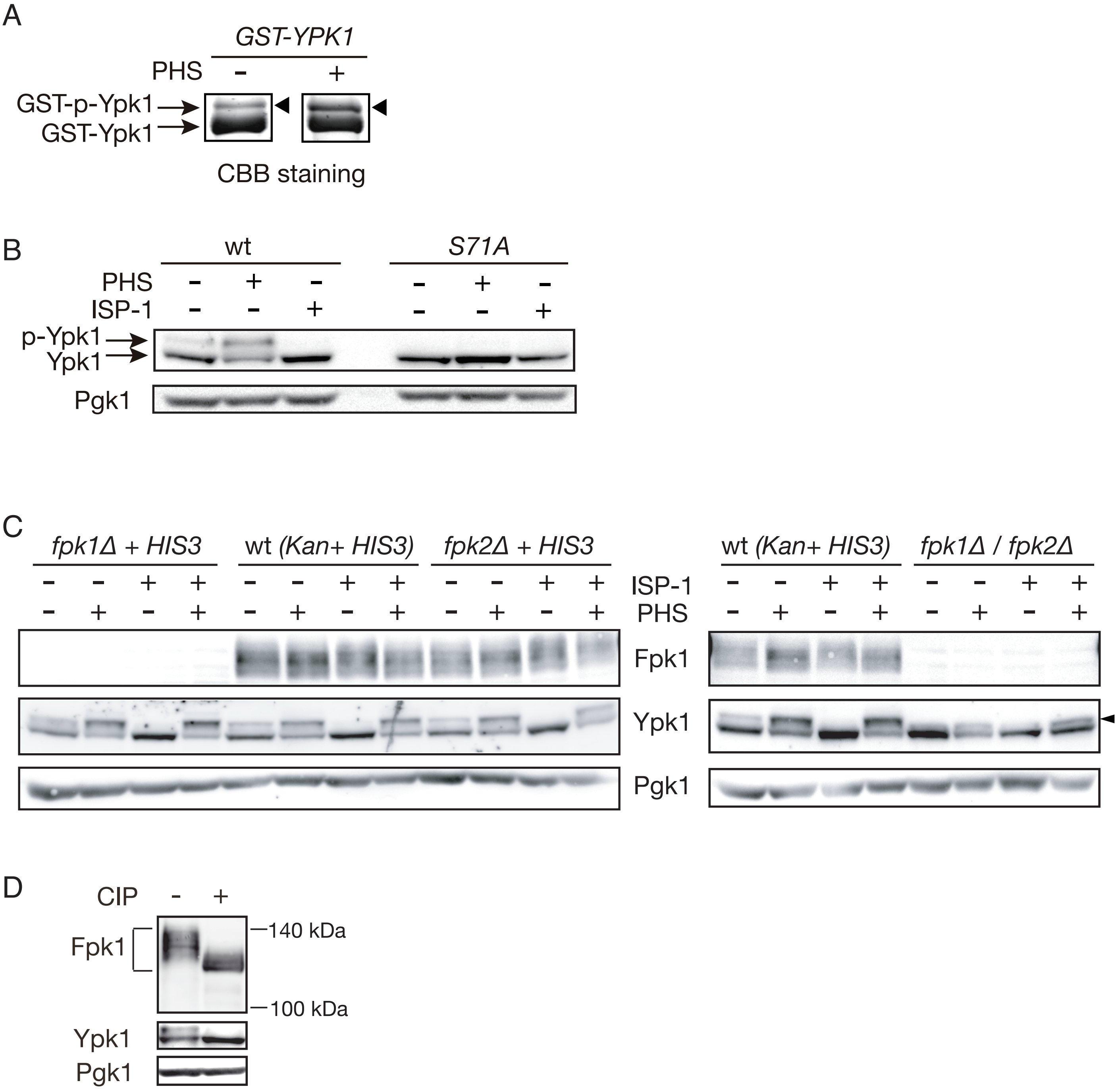

A

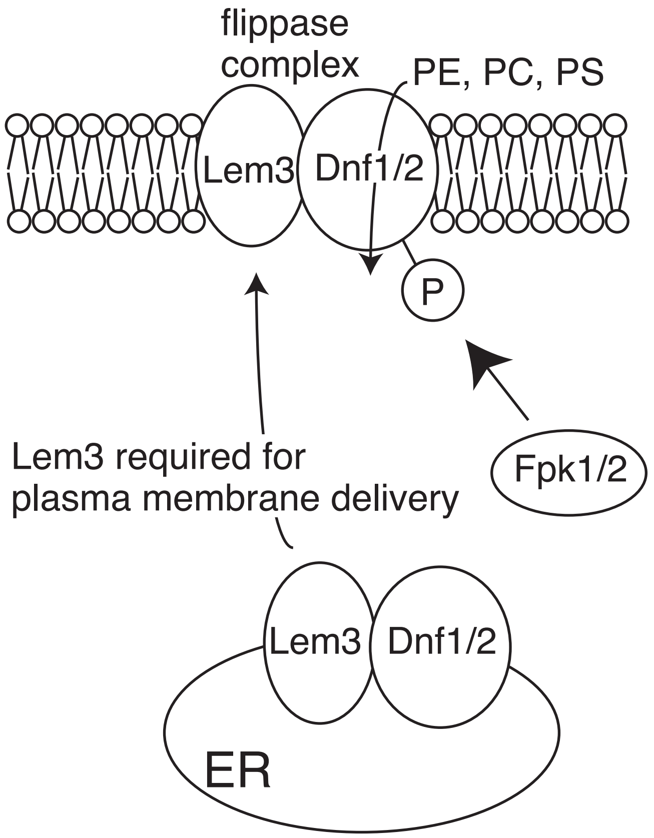
flippase
complex
PE, PC, PS
Lem3
Dnf1/2
P
Fpk1/2
Lem3 required for
plasma membrane delivery
Lem3
Dnf1/2
ER

B

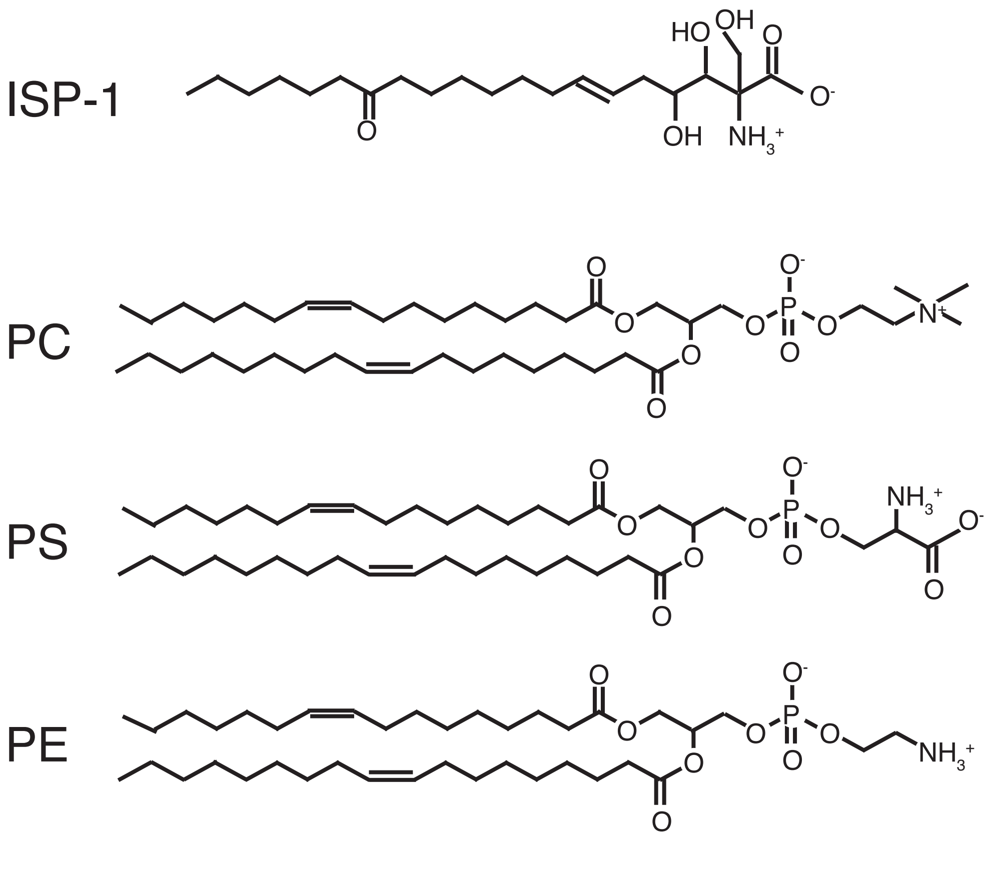
ISP-1
PC
PS
PE

Supplemental Figure S4

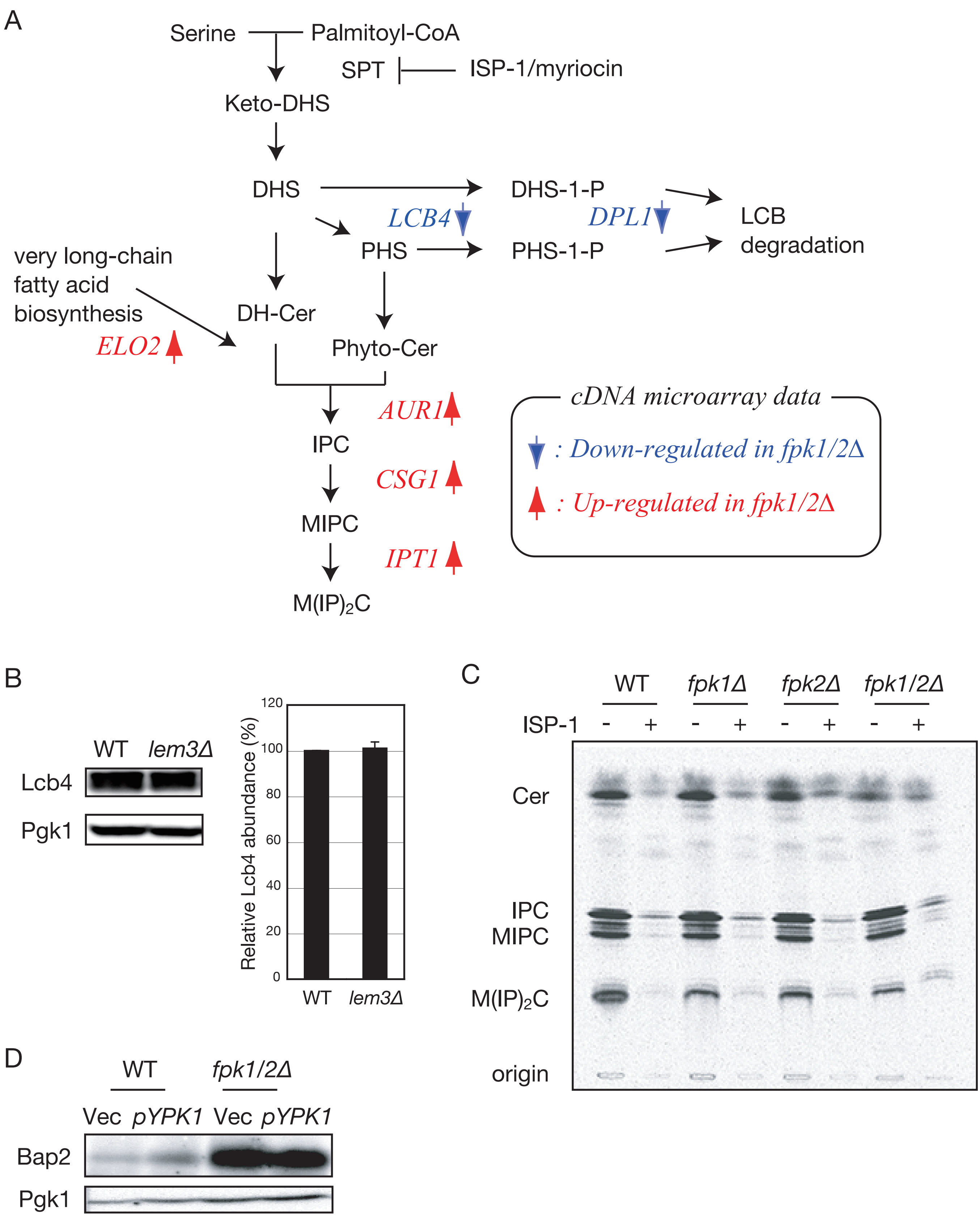

Supplemental Figure S5

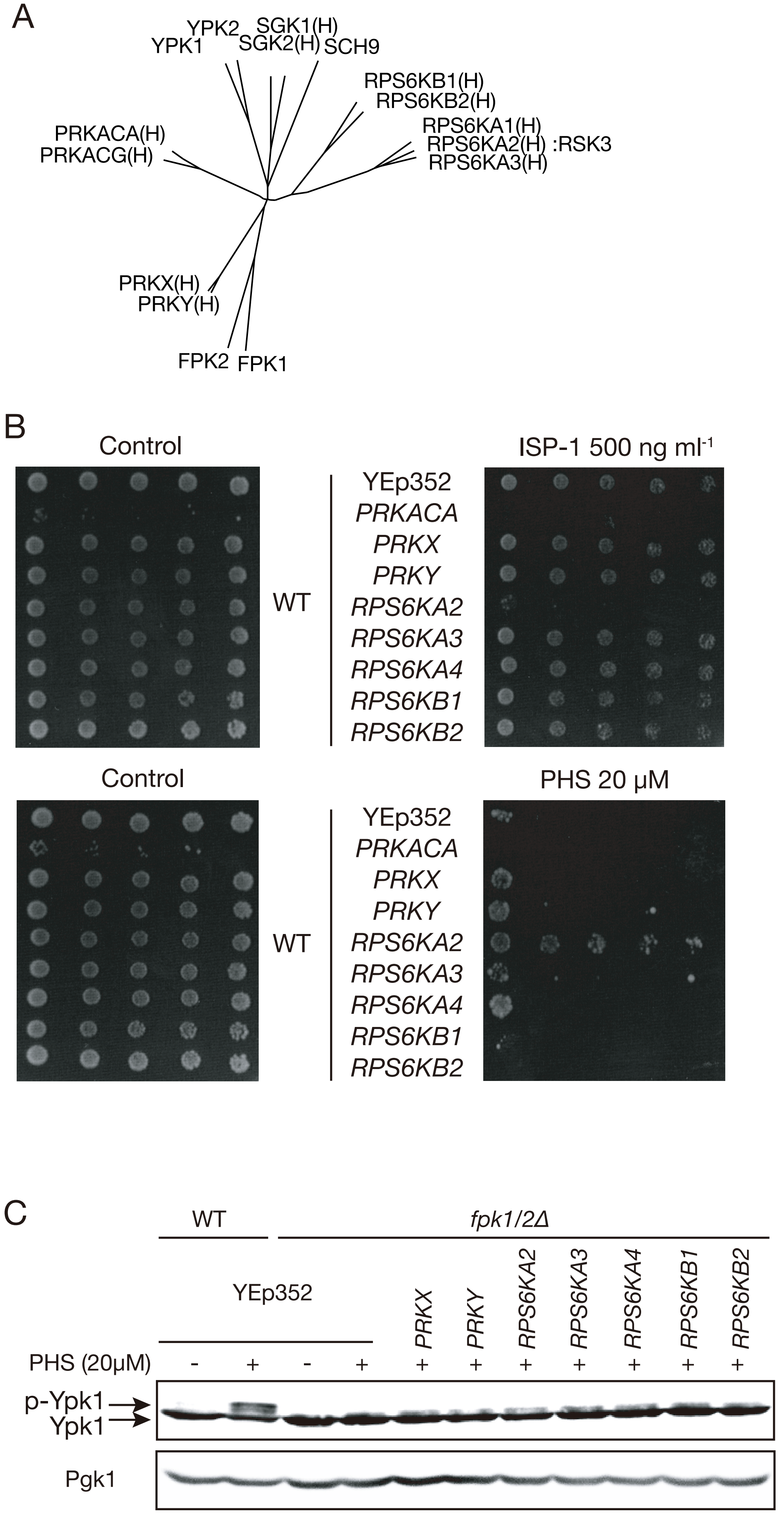

A

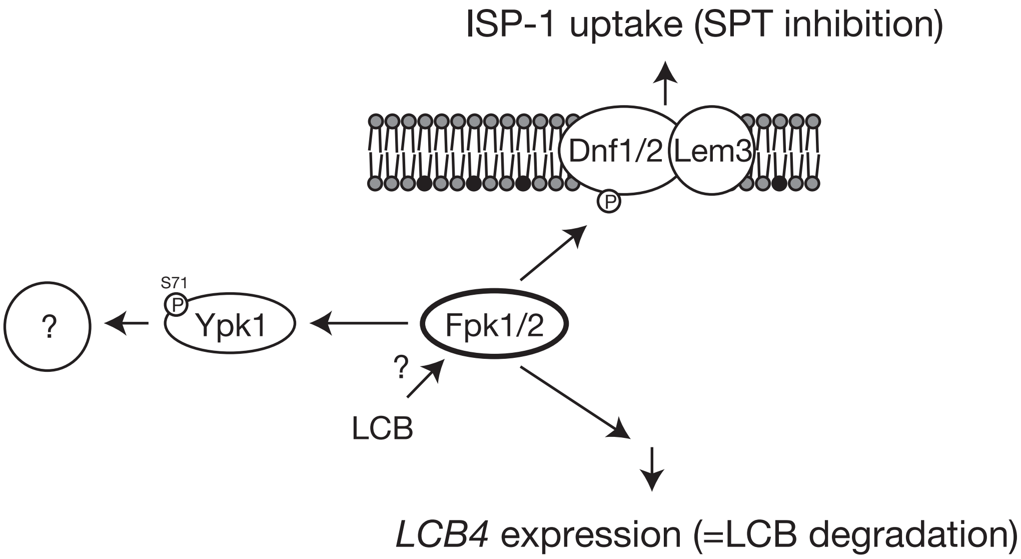
ISP-1 uptake (SPT inhibition)
Dnf1/2
Lem3
P
S71
P
Ypk1
?
Fpk1/2
?
LCB
LCB4 expression (=LCB degradation)

B

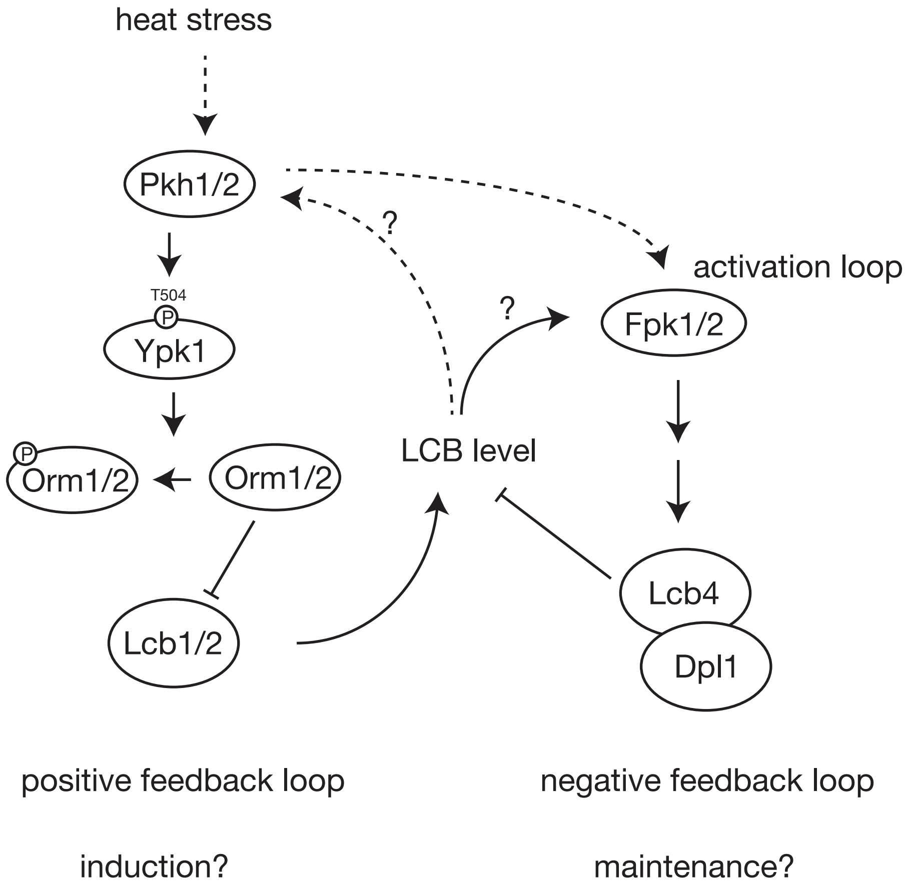
heat stress
Pkh1/2
?
activation loop
T504
P
Ypk1
?
Fpk1/2
P
Orm1/2
Orm1/2
LCB level
Lcb1/2
Lcb4
Dpl1
positive feedback loop
negative feedback loop
induction?
maintenance?

Supplemental Table S1 DIR screening

ORF	ISP-1 R		Sequence	ACC#	Superfamily	Group	Family	Specifi	Description
YBR028C	0		**YBR028C**	CAA84970	Protein kinase	AGC	AKT		YBR028C/YBR0312 Serine/threonine protein kinase with similarity to Ypk2p/Ykr2p and Ypk1p.
YKL126W	-2	DIV1	**YPK1/SLI2**	CAA81967	Protein kinase	AGC	AKT		YPK1/YKL126W Serine/threonine protein kinase with similarity to protein kinase C.
YMR104C	0		**YPK2**	CAA89740	Protein kinase	AGC	AKT		YPK2/YKR2/YM9718.03/YMR104C Serine/threonine protein kinase with similarity to Ypk1p.
YGR092W	-2	DIV2	**DBF2**	CAA97095	Protein kinase	AGC	NDR		DBF2/G4643/YGR092W Serine/threonine protein kinase Similar to Dbf20p, required for events in anaphase/telophase.
YPR111W	0		**DBF20**	AAB05204	Protein kinase	AGC	NDR		DBF20/P8283.6/YPR111W Cell cycle protein kinase Similar to Dbf2p, involved in termination of M-phase.
YJL164C	0		**TPK1**	CAA89459	Protein kinase	AGC	PKA		TPK1/PKA1/SRA3/PK25/J0541/YJL164C cAMP-dependent protein kinase 1, catalytic chain.
YPL203W	0		**TPK2**	CAA97917	Protein kinase	AGC	PKA		TPK2/PKA2/YKR1/P1855/YPL203W/PKA3 cAMP-dependent protein kinase 2, catalytic chain.
YKL166C	0		**TPK3**	CAA82008	Protein kinase	AGC	PKA		TPK3/PKA3/YKL630/YKL166C cAMP-dependent protein kinase 3, catalytic chain.
YDR466W	1		**PKH3**	AAB64902	Protein kinase	AGC	PKA-Rel	Yeast	YDR466W/D8035.10 Protein kinase activator of Pkc1-mitogen-activated protein kinase pathway
YDR490C	-1		**PKH1**	AAB64917	Protein kinase	AGC	PKA-Rel	Yeast	YDR490C/D8035.33/PKH1 Serine/threonine protein kinase functions similarly to mammalian 3-phosphoinositide-dependent protein kinase; phosphorylates and activates Ypk1p
YOL100W	-1		**PKH2**	CAA99113	Protein kinase	AGC	PKA-Rel	Yeast	YOL100W/HRC1081/O0784/PKH2 Serine/threonine protein kinase of unknown function.
YFL033C	0		**RIM15**	CAA04486	Protein kinase	AGC	RIM15/C	Yeast	RIM15; YFL033C Serine/threonine protein kinase; positive regulator of IME2 expression and of sporulation
YCR091W	1	DIR2	**KIN82/FPK2**	CAA42256	Protein kinase	AGC	S6K		KIN82/YCR1153/YCR091W Serine/threonine protein kinase of unknown function.
YNR047W	2	DIR1	**FPK1**	CAA96328	Protein kinase	AGC	S6K		YNR047W/N3449 Serine/threonine protein kinase of unknown function.
YGR080W	0		**TWF1**	CAA97082	Protein kinase	Atypical Pr	A6		TWF1; G4583; YGR080W Protein with similarity to human tyrosine kinase A6 PIR:A55922. Not in alignment.
YGL059W	0		**YGL059W**	CAA96762	Protein kinase	Atypical Pr	PDK		YGL059W/YGF9 Protein with similarity to human branched-chain alpha-ketoacid dehydrogenase kinase
YIL042C	-1		**YIL042C**	P40530	Protein kinase	Atypical Pr	PDK		YIL042C/YIE2/YI9905.06c Related to mitochondrial branched-chain a-ketoacid (BCKD) and pyruvate dehydrogenase (PDH) kinases, which are protein-serine kinases
YDR122W	0		**KIN1**	CAA88675	Protein kinase	CAMK	AMPK		KIN1/YD9727.17/YDR122W Serine/threonine protein kinase, Similar to Kin2p and S. pombe KIN1.
YLR096W	0		**KIN2**	CAA97659	Protein kinase	CAMK	AMPK		KIN2/L8004.3/L2546/YLR096W Serine/threonine protein kinase, Similar to Kin1p and S. pombe KIN1.
YOR233W	1	DIR5	**KIN4**	CAA99453	Protein kinase	CAMK	AMPK		KIN4/KIN31/(KIN3)/O5220/YOR233W Serine/threonine protein kinase Similar to Kin1p and Kin2p, catalytic domain is most Similar to Snf1p.
YDR477W	2	DIR4	**SNF1**	AAB64904	Protein kinase	CAMK	AMPK		SNF1/CAT1/CCR1/PAS14/HAF3/D8035.20/YDR477W Serine/threonine protein kinase essential for derepression of glucose-repressed genes, acts with Snf4p.
YPL141C	0		**YPL141C**	AAB68219	Protein kinase	CAMK	AMPK		YPL141C/LPI5 Serine/threonine protein kinase with similarity to Kin4p.
YPL150W	1		**YPL150W**	CAA97855	Protein kinase	CAMK	AMPK		YPL150W/P2597 Serine/threonine protein kinase of unknown function. (Not in YPD listing)
YFR014C	0		**CMK1**	BAA09253	Protein kinase	CAMK	CAMK		CMK1/YFR014C Calcium/calmodulin-dependent serine/threonine protein kinase (CaM kinase), type I.
YOL016C	0		**CMK2**	CAA99015	Protein kinase	CAMK	CAMK		CMK2/O2325/YOL016C Calcium/calmodulin-dependent serine/threonine protein kinase (CaM kinase), type II.
YDL101C	0		**DUN1**	CAA98668	Protein kinase	CAMK	CAMK		DUN1/ORF2370/YDL101C Protein kinase necessary for induction of Rnr3p and DNA repair genes after DNA damage. Contains FHA domain.
YGL158W	2	DIR6?	**RCK1**	CAA96870	Protein kinase	CAMK	CAMK		RCK1/G1854/YGL158W Serine/threonine protein kinase with similarity to Cmk1p, Cmk2p, and Cmk3p. (Seq updated)
YLR248W	-1		**RCK2**	AAB67392	Protein kinase	CAMK	CAMK		RCK2/CMK3/CLK1/L9672.6/YLR248W Calcium/calmodulin-dependent serine/threonine protein kinase (CaM kinase).
YKL048C	2		**ELM1**	CAA81883	Protein kinase	CAMK	ELM	Yeast	ELM1/YKL261/YKL048C Serine/threonine protein kinase regulating pseudohyphal growth.
YER129W	1		**SAK1/PAK1**	AAC03227	Protein kinase	CAMK	ELM	Yeast	PAK1/SYGP-ORF45/YER129W Snf1 Activating Kinase
YGL179C	-1		**TOS3**	CAA96762	Protein kinase	CAMK	ELM	Yeast	YGL179C/BIE560/G1618 Protein kinase, related to and redundant with Elm1p and Pak1p in activating the SNF1 complex
YDR507C	0		**GIN4**	AAB64949	Protein kinase	CAMK	EMK/GIN4		GIN4/D9719.13/YDR507C Serine/threonine-protein kinase with similarity to Yc1024p, growth inhibitory protein.
YKL101W	0		**HSL1**	CAA81941	Protein kinase	CAMK	EMK/GIN4		HSL1/YKL453/YKL101W Serine/threonine protein kinase that interacts genetically with histone mutations.
YCL024W	0		**KCC4**	CAA42361	Protein kinase	CAMK	EMK/GIN4		YCL024W Kinase coordinate cell cycle progression with the organization of the peripheralcytoskeleton
YOR351C	0		**MEK1**	CAA99680	Protein kinase	CAMK	Other		MEK1/MRE4/O6357/YOR351C Serine/threonine protein kinase required for meiotic recombination. Contains forkhead-associated (FHA) nuclear signalling domian.
YMR291W	1		**ESC1**	CAA56800	Protein kinase	CAMK	Other		YMR291W/ORF530348 Serine/threonine protein kinase of unknown function.
YKL116C	0		**PRR1**	CAA81955	Protein kinase	CAMK/EMI	Unique	Yeast	YKL116C/YKL516 Protein kinase with a possible role in MAP kinase signaling in the pheromone response pathway
YHR135C	2	DIR9	**YCK1**	AAB68417	Protein kinase	CKI	CKI		YCK1/CKI2/YHR135C Casein kinase I isoform.
YER123W	0		**YCK3**	AAC03221	Protein kinase	CKI	CKI		YCK3/CKI3/YER123W Casein kinase I isoform.
YPL031C	-2	DIV3	**PHO85**	AAB68188	Protein kinase	CMGC	CDK		PHO85/P7102 18A/YPL031C Cyclin-dependent protein kinase that interacts with cyclin Pho80p to regulate phosphate pathway. PSTAIRE in kinase domain III.
YJL106W	0		**IME2**	CAA89401	Protein kinase	CMGC	CLK		IME2/SME1/J0817/YJL106W STK and positive regulator of sporulation genes that is essential for initiation of meiosis. TAY in kinase domain VIII (activation loop).
YLL019C	0		**KNS1**	CAA97468	Protein kinase	CMGC	CLK		KNS1/L1224/YLL019C Serine/threonine protein kinase of unknown function. (Similar to CLK_h)
YJL141C	1		**YAK1**	CAA89437	Protein kinase	CMGC	CLK		YAK1/YJL141C Serine/threonine protein kinase that suppresses loss of Tpk1p + Tpk2p + Tpk3p. (Similar to KA23_sp, MNB_dm, MNB_h)
YMR216C	0		**YMR216C**	CAA89931	Protein kinase	CMGC	CLK		YMR216C/YM8261.10 Putative serine/threonine protein kinase, involved in phosphorylation of Npl3p, similar to Cdc31p. (Similar to DSK1_sp, SRPK1_ce, U52111_h).
YNL307C	0		**MCK1**	CAA96236	Protein kinase	CMGC	GSK		MCK1/(YPK1)/N0392/YNL307C Serine/threonine/tyrosine protein kinase (meiosis and centromere regulatory kinase), positive regulator of meiosis and spore formation.
YMR139W	1		**MDS1**	CAA87353	Protein kinase	CMGC	GSK		MDS1/RIM11/GSK3/YM9375.08/YMR139W Serine/threonine protein kinase, required for induction of IME2 by Ime1p, homolog of mammalian GSK3.
YDL079C	0		**MRK1**	CAA98645	Protein kinase	CMGC	GSK		MRK1/D2459/D2461/YDL079C Serine/threonine protein kinase with similarity to Mds1p.
YOL128C	0		**YGK3**	CAA99147	Protein kinase	CMGC	GSK		YOL128C/O0530/ORF1209713 Serine/threonine protein kinase activates transcription of stress responsive genes
YBL016W	0		**FUS3**	CAA84835	Protein kinase	CMGC	MAPK	ERK	FUS3/DAC2/YBL0303/YBL03.21/YBL016W Serine/threonine protein kinase of the MAP kinase family required for cell cycle arrest and for cell fusion during mating. TEY in kinase domain VIII.
YLR113W	-2	DIV5	**HOG1**	CAA97680	Protein kinase	CMGC	MAPK	p38	HOG1/SSK3/L9354.2/L2931/YLR113W Serine/threonine protein kinase of MAP kinase (MAPK) family, involved in high-osmolarity signal transduction pathway. TGY in kinase domain VIII.
YGR040W	-1		**KSS1**	CAA97038	Protein kinase	CMGC	MAPK	ERK	KSS1/G4149/YGR040W Serine/threonine protein kinase of the MAP kinase family, redundant with Fus3p for cell cycle arrest. TEY in kinase domain VIII (activation loop).
YHR030C	-2	DIV4	**SLT2/MPK1**	AAB68912	Protein kinase	CMGC	MAPK	ERK	SLT2/MPK1/SLK2/BYC2/YHR030C Serine/threonine protein kinase of MAP kinase family involved in the cell wall integrity pathway. TEY in kinase domain VIII.
YKL161C	0		**YKL161C**	CAA82003	Protein kinase	CMGC	MAPK	ERK	YKL161C/YKL615 Serine/threonine protein kinase, involved in protection against oxidative stress
YKL139W	2	DIR3	**CTK1**	CAA81980	Protein kinase	CMGC	Other		CTK1/YKL139W Carboxyl-terminal domain (CTD) kinase alpha subunit, CDK that phosphorylates C-terminal domain of RNA polymerase II large subunit. PITSIRE in kinase domain III.
YLR253W	0		**YLR253W**	AAB67388	Protein kinase	Microbial F	ABC1	sc	YLR253W/L9672.2/YL53 Protein with weak similarity to Abc1p
YPL109C	0		**YPL109C**	AAB68252	Protein kinase	Microbial F	ABC1	sc	YPL109C; LPH17 Protein of unknown function
YGR262C	-2	DIV10	**BUD32**	CAA97291	Protein kinase	Microbial F	YGR262	sc	YGR262C/G9334 Protein with similarity to apple tree calcium/calmodulin-binding protein kinase PIR:JQ2251.
YGR188C	-2	DIV9	**BUB1**	CAA97214	Protein kinase	Other	BUB		BUB1/G7542/YGR188C Serine/threonine protein kinase and checkpoint protein required for cell cycle arrest in response to loss of microtubule function. (N-terminus Similar to MAD3_sc)
YBR274W	0		**CHK1**	CAA85237	Protein kinase	Other	CHK1		YBR274W/YBR1742 Checkpoint kinase, Regulats inhibitory Cdk phosphorylation of Pds1
YIL035C	-1		**CKA1**	P15790	Protein kinase	Other	CKII		CKA1/YIL035C Casein kinase II, catalytic (alpha) subunit.
YOR061W	-1		**CKA2**	CAA99254	Protein kinase	Other	CKII		CKA2/O2810/YOR061W Casein kinase II, catalytic (alpha) subunit.
YDR283C	0		**GCN2**	AAB64461	Protein kinase	Other	EIFK		GCN2/AAS1/D9954.16/YDR283C Serine/threonine protein kinase that regulates initiation of translation by phosphoryation of eIF2alpha (Sui2p) (Similar to EIF2aK_r, HRI_r)
YJL165C	0		**HAL5**	CAA89460	Protein kinase	Other	NPR/HAI	Yeast	HAL5/J0531/YJL165C Serine/threonine protein kinase involved in salt and pH tolerance.
YNL183C	0		**NPR1**	CAA96076	Protein kinase	Other	NPR/HAI	Yeast	NPR1/N1631/YNL183C Serine/threonine protein kinase involved in regulating transport systems for nitrogen nutrients under conditions of nitrogen catabolite derepression.
YKL198C	0		**PTK1**	CAA82043	Protein kinase	Other	NPR/HAI	Yeast	PTK1/YKL198C/STK1/KKT8/(POT1) Serine/threonine protein kinase activator of low-affinity, low-capacity polyamine transport (Frame shift corrected)
YCR008W	0		**SAT4**	CAA42325	Protein kinase	Other	NPR/HAI	Yeast	SAT4/YCR101/YCR046/YCR008W Protein with similarity to Npr1p protein kinase.
YDL025C	0		**YDL025C**	CAA98584	Protein kinase	Other	NPR/HAI	Yeast	YDL025C/D2810 Protein with similarity to protein kinase Npr1p.
YDL214C	0		**YDL214C**	CAA98792	Protein kinase	Other	NPR/HAI	Yeast	YDL214C/D1014 Serine/threonine protein kinase with similarity Npr1.

YJR059W	1		**PTK2**	CAA89587	Protein kinase	Other	NPR/HAI	Yeast	YJR059W/J1725/PTK2/STK2 Putative serine/threonine protein kinase required for high-affinity polyamine transport
YKL168C	1		**YKL168C**	CAA82010	Protein kinase	Other	NPR/HAI	Yeast	YKL168C/YKL632/KKQ8 Serine/threonine protein kinase of unknown function.
YOR267C	0		**HRK1**	CAA99490	Protein kinase	Other	NPR/HAI	Yeast	YOR267C/O5420 Serine/threonine kinase protein kinase with a role in ion homeostasis
YAL017W	0		**PSK1**	AAC04940	Protein kinase	Other	PIM		YAL017W/YAL002/FUN31 PAS kinase involved in the control of sugar metabolism and translation
YOL045W	-1		**YOL045W**	CAA99051	Protein kinase	Other	PIM		YOL045W/02034/YOL044W PAS domain-containing Serine/threonine Kinase
YHR082C	0		**KSP1**	AAB68896	Protein kinase	Other	RAN	Yeast	KSP1/YHR082C Serine/threonine kinase, has similarity to casein kinase II and other serine/threonine protein kinases.
YPL026C	0		**SHA3**	AAB68161	Protein kinase	Other	RAN	Yeast	SHA3/SKS1/LPB5/YPL026C Serine/threonine protein kinase that suppresses the growth defect of snf3 mutants on low glucose
YDR247W	0		**YDR247W**	CAA89733	Protein kinase	Other	RAN	Yeast	YDR247W/YD8419.14 Protein kinase involved in G1/S transition
YGL083W	0		**SCY1**	CAA96788	Protein kinase	Other	SCY1	sc	SCY1/G3197/YGL083W Protein involved in suppression of a GTPase mutant
YGL180W	0		**ATG1**	CAA96892	Protein kinase	Other	UNC		YGL180W/G1615/APG1/AUT3 Serine/threonine protein kinase involved in induction of autophagy after nutrient limitation (Similar to UNC-51 ce, PLO1 sp)
YJL187C	0		**SWE1**	CAA89482	Protein kinase	Other	WEE		SWE1/J0406/YJL187C Serine/tyrosine dual-specificity protein kinase able to phosphorylate Cdc28p on tyrosine and inhibit its activity. (Similar to Wee1 sp, and MLK h)
YBR059C	2	DIR11	**AKL1**	CAA85002	Protein kinase	Other	YNL020(	sc, ce	YBR059C/YBR0419/AKL1 Serine/threonine protein kinase of unknown function.
YIL095W	1		**PRK1**	P40494	Protein kinase	Other	YNL020(	sc, ce	YIL095W/(PAK1)/PRK1 Serine/threonine protein kinase involved in regulation of actin cytoskeleton organization
YNL020C	1	DIR10	**ARK1**	CAA95882	Protein kinase	Other	YNL020(	sc, ce	YNL020C/N2823/ARK1 Serine/threonine protein kinase with probable involvement in cytoskeletal function
YPL236C	0		**YPL236C**	CAA97954	Protein kinase	Other	YPL236	sc	YPL236C/P1057 Protein of unknown function. - No GXG (Similar to C3H1.13 sp) (Not in YPD)
YAR018C	1		**KIN3**	AAC04964	Protein kinase	STE	NEK		KIN3/NPK1/FUN52/YAR018C Serine/threonine protein kinase, null mutation has no phenotype. TTY in kinase domain VIII (activation loop).
YJL095W	0		**BCK1**	CAA89389	Protein kinase	STE	STE11		BCK1/(SLK1)/SSP31/LAS3/SAP3/J0906/YJL095W Serine/threonine protein kinase of the MEKK family, involved in the cell wall integrity pathway.
YNR031C	-2	DIV7	**SSK2**	CAA96311	Protein kinase	STE	STE11		SSK2/N3276/YNR031C Map kinase kinase kinase of the high osmolarity signal transduction pathway.
YCR073C	0		**SSK22**	CAA42271	Protein kinase	STE	STE11		SSK22/YCR073C Map kinase kinase kinase with strong similarity to Ssk2p, participates in the high osmolarity signal transduction pathway.
YLR362W	2	DIR7?	**STE11**	CAA37522	Protein kinase	STE	STE11		STE11/L8039.10/YLR362W Serine/threonine protein kinase of the MEKK family, component of the pheromone pathway and a pathway regulating filamentous growth.
YNL298W	1	DIR8	**CLA4**	CAA96216	Protein kinase	STE	STE20		CLA4/ERC10/N0450/YNL0450/YNL298W Serine/threonine protein kinase required for cytokinesis, has similarity to Ste20p.
YDR523C	0		**SPS1**	AAB64963	Protein kinase	STE	STE20		SPS1/D9719.27/YDR523C Serine/threonine protein kinase involved in middle/late stage of meiosis.
YHL007C	0		**STE20**	AAB69747	Protein kinase	STE	STE20		STE20/YHL007C Serine/threonine protein kinase of the pheromone pathway, also participates in pathway regulating filamentous growth.
YOL113W	1		**SKM1**	CAA99132	Protein kinase	STE	STE20		YOL113W/HRA655/O0722/SKM1 Serine/threonine protein kinase with similarity to Ste20p.
YOR231W	0		**MKK1**	CAA99451	Protein kinase	STE	STE7		MKK1/SSP32/O5095/YOR231W Serine/threonine protein kinase of the MAP kinase kinase (MEK) family involved in cell wall integrity pathway.
YPL140C	0		**MKK2**	AAB68220	Protein kinase	STE	STE7		MKK2/SSP33/LPI6/YPL140C Serine/threonine protein kinase of the MAP kinase kinase (MEK) family involved in cell wall integrity pathway. (Sequence updated)
YJL128C	-2	DIV6	**PBS2**	CAA89423	Protein kinase	STE	STE7		PBS2/HOG4/SFS4/SSK4/OSR1/J0699/YJL128C Tyrosine protein kinase of the MAP kinase kinase (MEK kinase) family, essential component of the high-osmolarity signal transduction pathway.
YDL159W	-1		**STE7**	CAA98732	Protein kinase	STE	STE7		STE7/D1525/YDL159W Serine/threonine/tyrosine protein kinase of MAP kinase kinase (MEK) family, component of the pheromone pathway and a pathway regulating filamentous growth.
YJL057C	0		**IKS1**	CAA89348	Protein kinase	Yeast PK	Unique	Yeast	IKS1/J1143/YJL057C Probable serine/threonine protein kinase
YGR052W	0		**FMP48**	CAA97052	Protein kinase	Yeast PK	Unique	Yeast	YGR052W/G4329 Protein of unknown function, mitochondrial
YKL171W	0		**YKL171W**	CAA82013	Protein kinase	Yeast PK	Unique	Yeast	YKL171W/YKL635 Serine/threonine protein kinase of unknown function. (Similar to none)
YLR006C	-2	DIV8	**SSK1**						Cytoplasmic response regulator; part of a two-component signal transducer that mediates osmosensing via a phosphorylation of Ssk2; required for mitophagy;
YJR066W	0		**TOR1**	CAA89594	Protein kinase-like	Inositol Kir	PI3K		TOR1/DRR1/J1803/YJR066W Phosphatidylinositol kinase(PI kinase) homolog involved in cell growth and sensitivity to the immunosuppressant rapamycin.

? DIR6 strain exhibited strong resistance with strong LCB expression. However, the phenotype was not rescued by complementation of RCK1, indicating that some other gene was affected in this mutant strain to confer the phenotype.

? DIR7 strain exhibited strong resistance without basal LCB overexpression. Genotyping showed STE11 gene was not disrupted, indicating other mutation is responsible for the phenotype.

Supplemental Table S2 DNA Microarray (fpk1/2d vs wt)

Systematic ID	Gene name	Other name	S-B*norm(WT)	S-B(fpk1/2d)	FOLD fpk1/2d/wt(norm)
YBR068C	BAP2		660	2160	3.51
YOR224C	RPB8		4790	12001	2.69
YDR072C	IPT1	SYR4	3014	7500	2.67
YKL004W	AUR1		4057	9427	2.49
YCR034W	FEN1	ELO2|GNS1|VBM2	4137	9540	2.48
YDL061C	RPS29B	YS29B	7381	15797	2.30
YPL057C	SUR1	BCL21|CSG1|LPE15	1649	3101	2.02
YHR021C	RPS27B		12681	23710	2.01
YPR143W	RRP15		1622	310	0.21
YKL082C	RRP14		743	166	0.24
YOR171C	LCB4		479	107	0.24
YOL041C	NOP12		909	214	0.25
YJL148W	RPA34		2580	690	0.29
YDL136W	RPL35B	SOS2	23429	6354	0.29
YER002W	NOP16		1074	294	0.29
YBL027W	RPL19B		6595	1810	0.29
YPL090C	RPS6A		27071	7571	0.30
YBR247C	ENP1	MEG1	466	131	0.30
YLR009W	RLP24		1395	415	0.32
YHR052W	CIC1	NSA3	2524	797	0.34
YPL036W	PMA2		1514	500	0.35
YGR148C	RPL24B	RPL30B	20731	6790	0.35
YHR065C	RRP3		880	292	0.36
YNL162W	RPL42A		13153	4423	0.36
YPL217C	BMS1		924	316	0.37
YPR137W	RRP9		387	133	0.37
YBR191W	RPL21A	URP1	9994	3582	0.38
YER090W	TRP2		1382	485	0.38
YDR477W	SNF1/DIR4	CAT1|CCR1|GLC2|HAF3	474	173	0.39
YDR146C	SWI5		350	128	0.39
YGL008C	PMA1		46142	17028	0.40
YDR208W	MSS4/SLI6		310	115	0.40
YMR104C	YPK2	YKR2	352	133	0.41
YNR053C	NOG2		841	321	0.41
YIL069C	RPS24B	RPS24EB	8330	3183	0.41
YLR262C-A	TMA7	RBF7	2211	856	0.42
YGR280C	PXR1	GNO1|PINX1	798	311	0.42
YPR010C	RPA135	RPA2|RRN2|SRP3	1578	645	0.44
YPL160W	CDC60	LeuRS	3992	1642	0.44
YDR264C	AKR1/DIR10		734	303	0.44
YPL125W	KAP120		578	239	0.44
YCL031C	RRP7		642	268	0.45
YDL184C	RPL41A	RPL47A	1893	796	0.45
YIL105C	SLM1	LIT2	1511	637	0.45
YKL014C	URB1	NPA1	377	160	0.45
YGL026C	TRP5		7585	3240	0.46
YJL176C	SWI3	TYE2	433	186	0.46
YOR004W	UTP23		1932	832	0.46
YBL072C	RPS8A		29188	12702	0.47
YER074W	RPS24A	RPS24EA	31411	13673	0.47
YDL060W	TSR1		459	202	0.47
YJR059W	PTK2	STK2	522	226	0.47
YKL006W	RPL14A		9428	4209	0.48
YOR206W	NOC2		1567	702	0.48
YDR294C	DPL1	BST1	404	182	0.48
YLL008W	DRS1		2205	1007	0.49
YER102W	RPS8B		40414	18563	0.49
YDR465C	RMT2		536	247	0.50
YNL096C	RPS7B		7940	3666	0.50
